# Subject-Specific Cardiovascular Signatures Revealed by Arterial Pulse Signals in Patients with Heart Diseases—Part I: General Cases

**DOI:** 10.3390/diagnostics16213579

**Published:** 2026-10-29

**Authors:** Md Mahfuzur Rahman, Mamun Hasan, Jennifer F. May, John M. Herre, Leryn J. Reynolds, Zhili Hao

**Affiliations:** 1 Department of Mechanical and Aerospace Engineering, Old Dominion University, Norfolk, VA 23529, USA; mrahm009@odu.edu (M.M.R.); mhasa004@odu.edu (M.H.); 2 Sentara Health Research Center, Virginia Beach, VA 23464, USA; jlfreema@sentara.com; 3 Department of Medicine, Division of Cardiology, Macon and Joan Brock Virginia Health Sciences at Old Dominion University, Norfolk, VA 23501, USA; jmherre@sentara.com (J.M.H.); lreynold@odu.edu (L.J.R.); 4 Advanced Heart Failure and Heart Transplantation, Sentara Norfolk General Hospital, Norfolk, VA 23507, USA; 5 School of Exercise Science, Old Dominion University, Norfolk, VA 23529, USA

**Keywords:** arterial pulse signal, arterial pulse waveform, harmonic amplitude, tactile sensor, time-frequency analysis, single-degree-of-freedom (SDOF), heart rate variability, respiration

## Abstract

**Background/Objectives:** This study evaluates whether cardiovascular (CV) parameters derived from noninvasive arterial pulse signals can capture subject-specific physiological characteristics and longitudinal changes in patients with diverse heart diseases. **Method:** Arterial pulse signals at the radial artery (RA) and carotid artery (CA) of each patient were successfully recorded from all 18 patients using micro-fabricated tactile sensors. Signal quality was screened before analysis, and the accepted signals were analyzed using a single-degree-of-freedom time–frequency (SDOF-TF) framework. Key CV parameters, including time-based indices—heart rate (HR), heart rate variability (HRV), respiration rate (RR), and respiration modulation (RM), as well as harmonic amplitudes and normalized arterial pulse waveform (APW)—were extracted from measurements at rest and post-exercise across three rehabilitation visits in 18 patients with heterogeneous CV conditions. Longitudinal changes in CV parameters within subjects and variability between subjects were assessed. **Results:** Compared with a healthy reference, the patients exhibited heterogeneous CV profiles, with lower HRV in all patients except one with AF, who showed elevated HRV, and generally reduced post-exercise CV responses. Higher-order harmonic amplitudes were attenuated at the RA, while CA-derived APW measurements showed greater sensitivity to systolic upstroke slope. Marked inter-subject variability was evident in at-rest CV parameters and 5-min post-exercise responses. Longitudinal CV responses varied across rehabilitation visits, potentially reflecting multiple contributing factors. **Conclusions:** The 100% pulse acquisition rate supports the feasibility of the tactile sensor technology for pulse measurement, and the combined sensor technology and SDOF-TF framework enable quantitative characterization of shared and individualized CV patterns in cardiac patients and their post-exercise recovery, including time-based indices, harmonic signatures, site-specific differences, and interactions among CV parameters. These findings warrant further investigation in larger cohorts with appropriate physiological reference measurements.

## 1. Introduction

Cardiovascular (CV) disease remains the leading cause of morbidity and mortality worldwide [1,2,3,4]. Effective clinical management requires not only accurate diagnosis but also continuous monitoring of CV function during disease progression, treatment, rehabilitation and recovery [5,6,7,8,9]. Conventional clinical assessments typically rely on multiple instruments, including electrocardiography (ECG), echocardiography, heart rate monitoring, blood pressure monitoring, and respiratory measurements, each providing only a partial description of CV physiology [10,11,12,13,14]. While ECG and echocardiography require specialized equipment and are relatively costly, blood pressure monitoring alone provides limited information about cardiac activity [10,11,12,15,16,17]. Consequently, comprehensive evaluation of CV function often requires measurements from multiple instruments [7,10,11,12,18]. However, each instrument may introduce device-specific errors, leading to inconsistent or non-comparable outcomes [5,6,7,19,20]. Such measurement variability can complicate the assessment of CV physiology and limit the reliability of multi-instrument evaluations, highlighting a fundamental challenge in achieving accurate, continuous, and routine CV monitoring.

Arterial pulse signals carry rich information related to cardiac activity, vascular properties, and cardiorespiratory coupling, thereby providing a unique opportunity for integrated CV assessment [21,22,23,24,25]. In principle, multiple CV features can be extracted from a single arterial pulse signal. However, though there has been more than a century of investigation since the introduction of arterial pulse analysis for CV assessment, its use remains largely confined to research settings [26,27]. Two major challenges have prevented arterial pulse measurements from translating into routine clinical and home-based practice.

First, acquiring arterial pulse signals with high fidelity using tactile sensors remains relatively difficult in practice [28,29]. The measured signal is highly sensitive to measurement conditions (i.e., sensor alignment, contact pressure, and individual anatomy) and motion artifacts (MAs), which include body motion and respiration [30,31,32,33,34,35]. All of these measurement factors can distort measured arterial pulse morphology and reduce measurement reliability. Second, existing signal-processing techniques are often unable to effectively separate the pulse-induced signal (the component of the measured signal that responds to the arterial pulse) from MAs and sensor noise [21,30] in a measured pulse signal. As a result, extracting reliable CV features from measured pulse signals remains challenging in practice.

To date, the challenge of acquiring pulse signals with high fidelity has been partially addressed through advances in micro-fabricated tactile sensors. These sensors, developed in various forms, improve the conformity of the sensor to the artery and thereby enhance pulse signal transmission [36,37]. In contrast, developing a signal-processing techniques capable of effectively removing MAs and sensor noise while preserving physiologically meaningful information (i.e., the pulse-induced signal) remains a critical challenge [21,30,38,39,40,41,42].

To address this challenge, we developed the single-degree-of-freedom time–frequency (SDOF-TF) method for the effective removal of MAs and sensor noise from measured pulse signals [21]. This method is built upon a mechanical model of MAs, where the tissue–contact–sensor (TCS) stack between the sensor and artery is modeled as a SDOF system for fully capturing the dynamic behavior of the TCS stack under the influence of MA during pulse measurements and the effect of MA on the TCS stack is quantified as a time-varying system parameter (TVSP) [43]. This method enables simultaneous extraction of multiple CV parameters from a single arterial pulse measurement, providing a unified and cost-effective approach for integrated CV assessment under both at-rest and post-exercise recovery conditions.

When applied to photoplethysmography (PPG) signals from a previously reported dataset of atrial fibrillation (AF) and non-AF patients, identified using gold-standard ECG recordings, the SDOF-TF method achieved 100% classification accuracy in distinguishing AF from non-AF subjects, outperforming other signal-processing techniques [24]. Several extracted CV parameters were identified as potential clinical markers contributing to this detection capability. More recently, the method was applied to three representative subjects [22]: a healthy individual, a heart-transplant (HTx) patient, and a percutaneous coronary intervention (PCI) patient with idiopathic pulmonary fibrosis (IPF). The analysis revealed both shared altered CV features across patients and distinct subject-specific characteristics, demonstrating the method’s ability to capture both disease-related patterns and individualized physiological signatures from pulse signals.

However, this prior study [22] was limited to a small number of representative cases and did not address the broader applicability of the method to a larger, heterogeneous patient population. In particular, substantial inter-individual variability may complicate interpretation when extending beyond case-based analysis, highlighting the need for systematic evaluation across a wider cohort.

In this paper, which constitutes Part I of our work, arterial pulse signals from 18 patients with diagnosed heart diseases were acquired using the same tactile sensor described in the prior study [22] and analyzed using the SDOF-TF method to extract multiple CV parameters for patient-specific characterization. Measurements were obtained across three visits during an outpatient cardiac rehabilitation program, enabling simultaneous assessment of multiple CV parameters and their longitudinal changes.

Rather than focusing on group-level statistics, this paper emphasizes subject-specific characterization and the longitudinal changes in CV parameters within each individual (i.e., individualized CV profiling). Part II will focus on seven patients whose arterial pulse signals exhibit distinctive patterns, warranting a separate, detailed investigation of the corresponding CV signatures. The objectives of this study are to: (1) compare CV parameter patterns in cardiac patients with those of a healthy reference, (2) investigate the degree of subject-specific variability, and (3) evaluate whether longitudinal measurements reveal individualized CV trajectories.

Through this work, we assess the potential of the tactile sensor and the SDOF-TF method for individualized CV assessment and monitoring. More broadly, this study explores the potential value of individualized CV profiling using arterial pulse signals, while recognizing that larger cohorts and appropriate physiological reference measurements are needed to establish clinical relevance and validity.

## 2. Materials and Methods

The micro-fabricated tactile sensor and its pulse measurements and the SDOF-TF method have been described in previous studies [21,22,24]. For completeness, a brief description of them is provided here to facilitate a better understanding of the content of this paper.

### 2.1. Arterial Pulse Signal Acquisition

Arterial pulse signals were acquired using the same micro-fabricated tactile sensor and measurement configuration described previously [21,22,24]. The sensor consists of a polydimethylsiloxane (PDMS) microstructure embedded with a resistive 5 × 1 transducer array. When placed above an artery with contact pressure, the sensor detects the displacement of the arterial wall. Measurements were performed by positioning two sensors over the radial artery (RA) and the carotid artery (CA) simultaneously. The pulse signal recorded by the sensor is the displacement response of the tissue–contact–sensor (TCS) stack, which lies between the sensor and the artery, to the underlying arterial pulse.

Because of the existence of the TCS stack and unavoidable MAs, which includes respiration and body motion, the measured signal reflects a transformed version of the true arterial pulse. The TCS stack acts as a harmonic-dependent mechanical filter, and its behavior can be modeled as a single-degree-of-freedom (SDOF) system [21]. MAs introduce low-frequency baseline drift (BD) and time-varying system parameters (TVSPs) of the TCS stack, perturbing the TCS stack’s response. Thus, the TCS stack filters the arterial pulse harmonically, while MAs impose additional disturbances on this mechanical filtering process.

### 2.2. SDOF-TF Method for Time–Frequency Analysis of Measured Pulse Signals

Measured pulse signals are inherently nonstationary due to HRV and MAs. To accurately extract CV parameters, the signals were analyzed using the previously developed time–frequency analysis framework based on an SDOF-TF method [21,24]. Within this framework, the arterial pulse signal is represented as a superposition of harmonics of the fundamental heart rate (HR) frequency. Time–frequency analysis yields instantaneous amplitude, instantaneous frequency (IF), and initial phase for each harmonic component. These instantaneous quantities provide the basis for extracting CV parameters with the effective removal of MAs and accompanying sensor noise.

From a single measured pulse signal, the following CV parameters were derived:

- Heart rate (HR) averaged from the first three harmonics.
- Heart rate variability (HRV) averaged from the first three harmonics, with the HRV of each harmonic being calculated as the root mean squared error (RMSE) of instantaneous HR relative to the average HR over the analyzed pulse signal period [22]. This measure is derived from instantaneous frequency (IF), where IF × 60 gives instantaneous HR, rather than from beat-to-beat intervals, as discussed in Section 4.
- Standard deviation of HRV normalized to HRV across the first three harmonics (S_HRV_/HRV).
- Respiration rate (RR) averaged from the first three harmonics.
- Respiration modulation (RM) of each of the first three harmonics.
- HRV induced by physiological factors (PFs), other than respiration, (HRV_PF_) averaged from the first three harmonics.
- Normalized arterial pulse waveform (APW) (relative to the pulse amplitude) with constant HR, where HRV is removed to avoid APW variation across pulse cycles.
- Normalized amplitude (*A_i_*) of the *i*th harmonic (relative to the amplitude of the first harmonic) of the 2nd~7th harmonics (*i* = 2~7).
- Normalized amplitude change (Δ*A_i_*) of the *i*th harmonic 5 min and 10 min post-exercise (relative to the corresponding at-rest value) of the 2nd~7th harmonics (*i* = 2~7).

Details about the extraction of the above parameters can be found in the literature [21,24]. RM has been proposed as an index of respiratory modulation of heart rate, which may be related to respiratory sinus arrhythmia (RSA) and parasympathetic activity in subjects with sinus rhythm [23,44]. However, its physiological interpretation requires caution in patients with cardiac arrhythmias, such as AF, in whom irregular ventricular interval variability may complicate interpretation, as illustrated by the only patient with AF in this study (CR019).

As discussed previously [22], time-based indices, including HR, HRV, RR, and RM, are quantitatively reliable under low-level MAs and can be compared between subjects and across visits. Similarly, normalized APW and *A_i_* of the higher harmonics (*i* = 2~7) can be compared qualitatively between subjects and across visits at the same arterial site. However, quantitative comparisons of these waveform-related measures require accounting for the TCS stack, which varies with measurement conditions, arterial sites, and across individuals. Accordingly, no quantitative indices of arterial stiffness were extracted from the measured pulse signals at either artery in this study.

As discussed previously [22], time-based indices, including HR, HRV, RR, and RM, are quantitatively reliable under low-level MAs and can be compared between subjects and across visits. However, their reliability depends on the measurement site and the level of MA. Because MAs are generally greater at the CA than at the RA, time-based indices are less reliable at the CA, where temporal variations induced by TVSPs can affect their estimates. HR is less sensitive to TVSP-induced temporal variations than HRV, RR, and RM and therefore remains relatively reliable at the CA despite the greater MA.

In contrast, normalized APW and the normalized amplitude *A_i_* of the higher harmonics (*i* = 2~7) are relatively insensitive to large MA and remain useful for waveform characterization at the CA. Nevertheless, these waveform-related parameters are appreciably affected by the TCS system parameters, which vary with measurement conditions, arterial sites, and across individuals. Accordingly, normalized APW and harmonic amplitude distributions are compared qualitatively within the same subject across visits, whereas direct inter-subject comparisons are not considered appropriate. No quantitative indices of arterial stiffness were extracted from the measured pulse signals at either artery in this study.

### 2.3. Study Population and Measurement Protocol

The measurement protocol for the 25 patient subjects was approved by the Eastern Virginia Medical School, Virginia Health Sciences IRB, at Old Dominion University (IRB Approval #19-04-FB-0100), and written informed consent was obtained from all participants. Patients diagnosed with various forms of CV disease were recruited from the outpatient cardiac rehabilitation program at Sentara Heart Hospital, where all patient measurements were conducted. Among the 25 recruited patients, 7 special-case patients—including a heart transplant (HTx) recipient and individuals with bradycardia, low dicrotic notch (DN), and intermittent APW changes—are presented in Part II of this work. The present study focuses on the remaining 18 patient subjects. In addition, one individual free from known CV disease was measured at Old Dominion University (ODU) under a separate protocol and included as a healthy reference (41 years old; male; BMI: 19.9) for comparison. The measurement protocol for the healthy reference was approved by the ODU Institutional Review Board (IRB #IRB24-166), and written informed consent was obtained. Due to institutional policy and applicable privacy regulations, individual-level patient data will not be shared with external investigators or repositories. These restrictions are necessary to protect patient confidentiality and comply with institutional governance of clinical data resources.

Pulse signal measurements were obtained from each patient during three visits scheduled on the same weekday in the first (Visit 1), second (Visit 2), and fourth (Visit 3) weeks of the outpatient cardiac rehabilitation program to enable longitudinal assessment of CV status. During each visit, measurements were obtained at-rest (pre-exercise) and at 5, 10, 20, 30, and 60 min post-exercise. It should be noted that between 30 and 60 min post-exercise, patient activity could not be strictly controlled, and almost all the patients engaged in behaviors such as phone use. This lack of control was not initially accounted for during data acquisition but was identified during subsequent analysis, where the 60 min post-exercise measurements exhibited substantially larger fluctuations compared to earlier time points. These fluctuations are therefore attributed to uncontrolled external influences rather than physiological recovery alone. Accordingly, the 60 min post-exercise data were excluded from the analysis presented in Section 3.

Two identical micro-fabricated tactile sensors were used to simultaneously acquire arterial pulse signals at the RA and CA. For all CA measurements, a PDMS layer was interposed between the sensor and the skin to improve conformity with the artery [29], thereby enhancing the quality of the acquired pulse signal. The healthy reference was measured during a single visit at ODU, with data collected at rest and at 5, 10, and 25 min post-exercise using the same sensing system. All recorded pulse signals were processed using the SDOF-TF-based algorithm to extract the same CV parameters for each patient. The healthy reference’s measurement at 25 min post-exercise was used for comparison with the patients’ measurements at 30 min post-exercise.

Of the 18 patients in this study, the mean age was 65.2 years (SD = 8.8), and the mean BMI was 32.07 (SD = 5.22). At the initial cardiac rehabilitation evaluation, the mean blood pressure was 126 mmHg systolic (SD = 16.46) and 72.77 mmHg diastolic (SD = 8.00). The mean HR was 72.11 bpm (SD = 9.58). Six patients had diastolic dysfunction and 5 had systolic dysfunction. The most recent NT-proBNP level prior to cardiac rehabilitation ranged from 31 to 9095 pg/mL. Sixteen patients had normal sinus rhythm (SR). Four patients demonstrated left bundle branch block on EKG, and the mean QRS duration was 115 ms (SD = 36.8).

Table 1 summarizes the baseline demographic and clinical characteristics of the 18 patient subjects and the healthy reference. Subject numbering is nonconsecutive because some individuals initially consented to participate but subsequently withdrew before completing any measurement visits. Additionally, not all subjects completed the third visit. All listed characteristics were recorded before the first visit. Note that CR039 (PCI with IPF) and the healthy reference are the same subjects as in [22].

### 2.4. Exercise Protocol and Intensity Assessment

Patients participated in an outpatient cardiac rehabilitation (CR) program at Sentara Heart Hospital. The exercise protocol, which included multiple types of exercise, varied among patients and visits, with the types of exercise performed during CR detailed in Supplementary S1 of the Supplementary Materials. Patients typically completed approximately three types of exercise per visit, although some completed four or five. The total exercise duration was at least 31 min and could extend to approximately 50 min, depending on each patient’s ability and the time available during the session.

Cardiac activity during exercise was continuously monitored using the Scottcare VersaCare wireless telemetry system with a three-lead ECG configuration (electrodes positioned at the right arm [RA], left arm [LA], and right leg [RL]). Exercise intensity was characterized using the maximum HR recorded by the telemetry system during exercise, which could occur during any exercise activity, depending on the patient’s exertion level, rather than necessarily at the end of the session. The Borg Rating of Perceived Exertion (RPE) scale (6–20) was also used in the CR program to assess perceived exertion, with the majority of recorded RPE scores falling within the range of 10–12.

The healthy reference underwent a separate exercise protocol at Old Dominion University (ODU), consisting of approximately 5 min of jumping and squatting exercises. The maximum HR was expected to occur at the end of the 5-min exercise protocol. Because HR was not recorded continuously during exercise for the healthy reference, the HR extracted from the pulse measurement at 1 min post-exercise was used to represent the maximum HR, as HR remained elevated at that time. Table 2 summarizes the at-rest HR and maximum HR in the 18 patients and the healthy reference, along with the absolute and normalized differences between maximum and at-rest HR. At-rest HR values were obtained using both a medical instrument (Mindray Passport 12) and the tactile sensor at the RA.

The absolute difference, HR_max_ − HR_sensor_, and normalized difference, ΔHR_max_/HR = (HR_max_ − HR_sensor_)/HR_sensor_, between the maximum HR recorded by the telemetry system and the at-rest HR measured at the RA using the tactile sensor were used as descriptive measures of the observed HR response during exercise. The normalized difference, ΔHR_max_/HR, was used as a relative indicator of exercise intensity. These measures were not adjusted for age or other factors that may influence HR, including CV disease, medication use, and differences in exercise protocols. The data also show that some patients taking beta blockers achieved normalized HR differences exceeding the 68% observed in the healthy reference. Thus, substantial HR responses during exercise were observed in patients both with and without beta-blocker use. However, given the heterogeneity of the cohort and the lack of adjustment for other influencing factors, these descriptive observations do not establish the independent effect of beta blockers on exercise-related HR responses.

### 2.5. Signal Quality Assessment and Usable Signal Segments

Sensor alignment and contact pressure were adjusted during pulse measurement to establish appropriate sensor–artery conformity and acquire a clear pulse signal with low MA and sensor noise. Pulse measurements at each time point lasted approximately 1 min. The effects of MA on the measured arterial pulse signal include BD and TVSP-induced distortion [42,43]. MAs cause time-varying displacement between the sensor and the underlying artery; accordingly, MAs are quantified in terms of BD. As a low-frequency component below 0.7 Hz, BD can be readily identified and removed using appropriate filtering. In contrast, TVSP-induced distortion affects the individual harmonics of the measured pulse signal and therefore cannot be readily removed using conventional frequency filtering.

Before signal processing, the measured signals were visually inspected to identify segments with relatively low BD fluctuations for subsequent analysis. The identified segments of all collected signals were then processed using the SDOF-TF method to recover the pulse-induced signal from measurements affected by TVSP-induced distortion and sensor noise. Following signal processing, the quality of the pulse-induced signal was visually assessed by examining its magnitude relative to the TVSP-induced distortion and sensor noise. An example of this comparison is provided in our previous study [21]. The corresponding comparisons are not presented for the 18 patients in this study or the seven patients in Part II. A measured signal was considered suitable for CV parameter extraction when the TVSP-induced distortion and sensor noise were much smaller than the pulse-induced signal. This assessment was qualitative; no quantitative signal-quality metric or predefined acceptance threshold was used.

While most usable RA and CA segments exceeded 30 s, segments from a few measurements were as short as approximately 20 s. For these shorter segments, padding was applied to facilitate CV parameter extraction. CA acquisition was generally more challenging than RA acquisition. In particular, obtaining a usable signal segment longer than 20 s was especially challenging for the subject with IPF (CR039) in this study and the subject with extremely high BMI (CR011) in Part II.

The quality of the tactile sensor measurements, the subsequent assessment of signal quality following signal processing, and the effectiveness of the SDOF-TF method in recovering the pulse-induced signal provide the technical foundation for reliable CV parameter extraction.

### 2.6. Analytical Strategy

Given the heterogeneity of the study population and the diversity of underlying CV conditions, the analytical approach emphasizes individualized characterization of CV parameters and their longitudinal changes within each subject, rather than population-level statistical inference. Three complementary levels of analysis were performed:

1. Subject-level characterization: Each subject’s CV parameter set was analyzed to identify distinctive physiological features and patterns, highlighting individual deviations from healthy reference profiles.
2. Longitudinal comparison: Changes in parameters across the three visits were evaluated to assess potential improvement, stability, or deterioration in CV function. The interpretation of longitudinal changes considered the reliability of each parameter, the measurement site, and the potential influence of MAs and TCS mechanical parameters. Small changes may reflect measurement uncertainty (i.e., measurement errors), physiological variability, or a combination of both. Larger changes were interpreted as potentially reflecting physiological changes, considering the technical foundation established for reliable CV parameter extraction using the SDOF-TF method and the quality of the recovered pulse-induced signals. However, because measurement errors were not quantitatively characterized, no predefined threshold was used to distinguish measurement-related variations from physiological changes. Quantitative longitudinal comparisons of time-based indices were restricted to the RA because these indices are less reliable at the CA owing to greater MAs and TVSP-induced temporal variations. Normalized APW and harmonic amplitude distributions were compared qualitatively within each subject across visits because of their dependence on TCS system parameters. This framework enables careful interpretation of temporal trends while accounting for parameter-specific measurement reliability and physiological dynamics.
3. Cross-subject comparison: Qualitative comparisons were conducted across subjects to identify recurring patterns among patients and distinctive CV features specific to individual patients.

Overall, this multi-level analytical framework integrates subject-level characterization, longitudinal evaluation, and qualitative cross-subject comparison to characterize individualized CV patterns and explore potential recurring patterns among patients with heterogeneous CV conditions.

Because measured APW morphology and harmonic amplitude distributions depend on the measurement instrument, measurements from the single healthy reference serve as device-specific baseline values for descriptive comparison with those of the CV patients, rather than as normative values representative of the healthy population. In addition, the post-exercise responses observed in the healthy reference were consistent with related findings reported in the literature, providing supporting context for interpreting these responses while not establishing population-level normative values.

ChatGPT-6 (OpenAI; GPT-6) was used to polish author-written manuscript text for grammar, clarity, and readability. All of the scientific content, data analysis, interpretation, and conclusions were determined by the authors.

## 3. Results

As observed in the previous study [22], CA measurements were heavily affected by MAs; therefore, only their normalized APW is included for comparison with the RA measurements. All analyses of HR, HRV, RR, and RM are based on RA measurements.

### 3.1. HR and HRV

**Figure 1:**
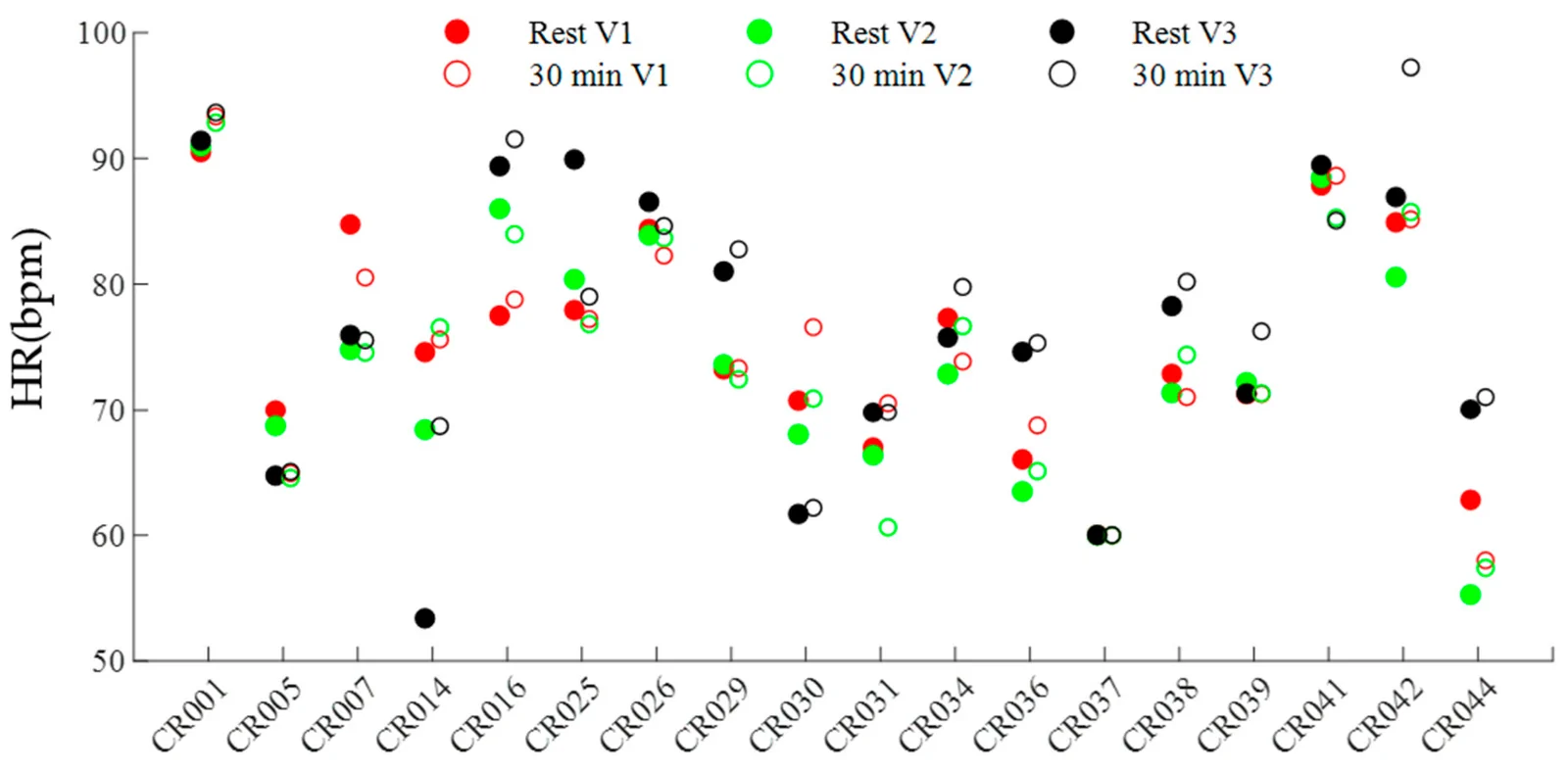
At-rest and 30 min post-exercise HR of the subjects (note: some subjects did not reveal a consistent increasing or decreasing trend of at-rest HR across the three visits).

As shown in Figure 1, at-rest HR varied across subjects, although most values remained within the normal range. Across visits, HR changes were generally moderate: some subjects exhibited increasing or decreasing trends, whereas others remained relatively stable. Notable cases included CR001 (chronic systolic HF, ICD) and CR041 (AF, PCI), who consistently exhibited high HR; CR014 (CABGX6) and CR044 (CABGX4), who showed pronounced longitudinal decreases; and CR037 (chronic systolic HF, CRT-D), who maintained an extremely stable HR. Given the technical foundation of the SDOF-TF method and the acceptable quality of the recovered pulse-induced signals, the large longitudinal changes in at-rest HR observed in some subjects are more likely to reflect physiological variation than measurement errors alone. Conversely, the extremely stable HR observed in CR037 suggests little physiological variation in at-rest HR across visits.

Figure 1 also compares the HR differences between at-rest and 30 min post-exercise across visits. Most patients exhibited slight to moderate changes across visits. Exceptions included CR014 (CABGX6), CR025 (MVR), and CR042 (CABG), who showed large HR differences at Visit 3, whereas CR037 remained unchanged across all visits. The large differences between at-rest HR and 30-min post-exercise HR observed across visits are more likely to reflect physiological variations in post-exercise HR responses, whereas the small differences observed in some subjects (e.g., CR001, CR037, and CR041) suggest little to no physiological variation in these responses across visits.

**Figure 2:**
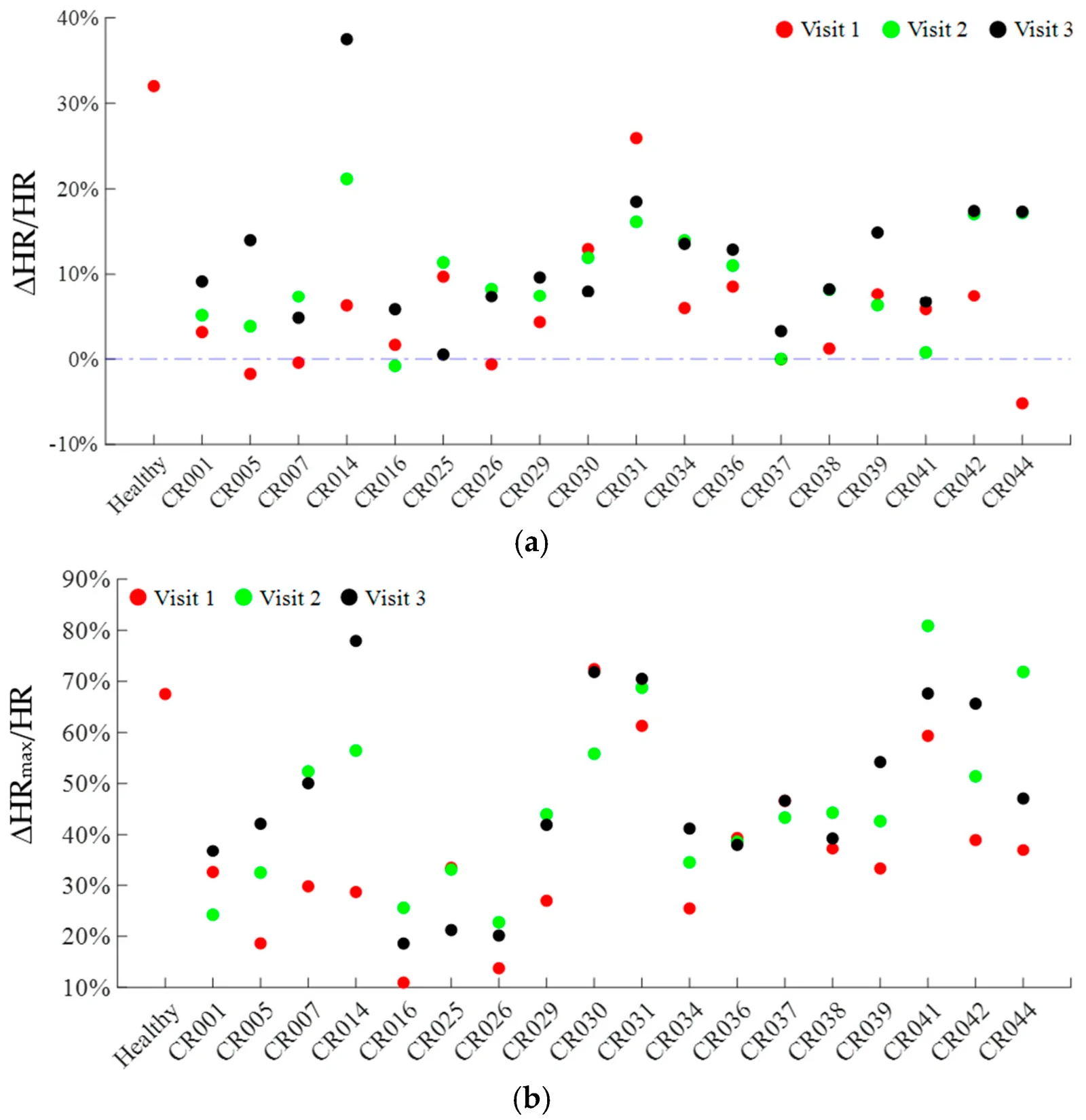
(**a**) Highest observed ΔHR/HR during post-exercise recovery of the subjects, occurring at 5 min post-exercise in most cardiac patients; (**b**) ΔHR_max_/HR during exercise, used as a relative indicator of exercise intensity.

Figure 2a plots the largest HR deviations relative to at-rest HR due to exercise, ΔHR/HR, of the subjects. The largest deviations from baseline typically occurred at 5 min post-exercise, although in some patients peak responses were observed at 10 min. A few patients exhibited lower HR post-exercise than at-rest HR, and for these, ΔHR/HR was taken at 5 min. Overall, the ΔHR/HR of all patients was much lower than that of the healthy reference, except for CR014 (CABGX6). Most subjects demonstrated a noticeable longitudinal increase in ΔHR/HR, with distinct patterns observed in several patients: CR025, CR030, and CR031 showed a notable decreasing trend; CR037 and CR041 showed minimal change; CR014 exhibited a large positive longitudinal change; and CR044 transitioned from a negative to a positive ΔHR/HR. Given that the signal-quality screening and SDOF-TF-based signal retrieval provide a technical basis for confidence in the extracted parameters, except for relatively small differences, the observed moderate and large inter-visit changes in ΔHR/HR of each subject across visits likely reflect true physiological variation, rather than measurement variability.

Figure 2b shows exercise intensity, defined as ΔHR_max_/HR during exercise, as reported in Table 2. Comparison of ΔHR/HR at 5 min post-exercise with ΔHR_max_/HR during exercise revealed heterogeneous relationships across subjects. For CR001, CR005, and CR014, the post-exercise ΔHR/HR generally varied in correspondence with exercise intensity across visits, whereas no clear relationship was apparent for CR030 and CR041. Notably, all five subjects were taking beta blockers, which may have influenced HR responses both during exercise and during post-exercise recovery. These observations suggest that the relationship between exercise intensity and post-exercise HR responses varied among subjects; however, the heterogeneous cohort and descriptive nature of this analysis preclude stronger conclusions.

**Figure 3:**
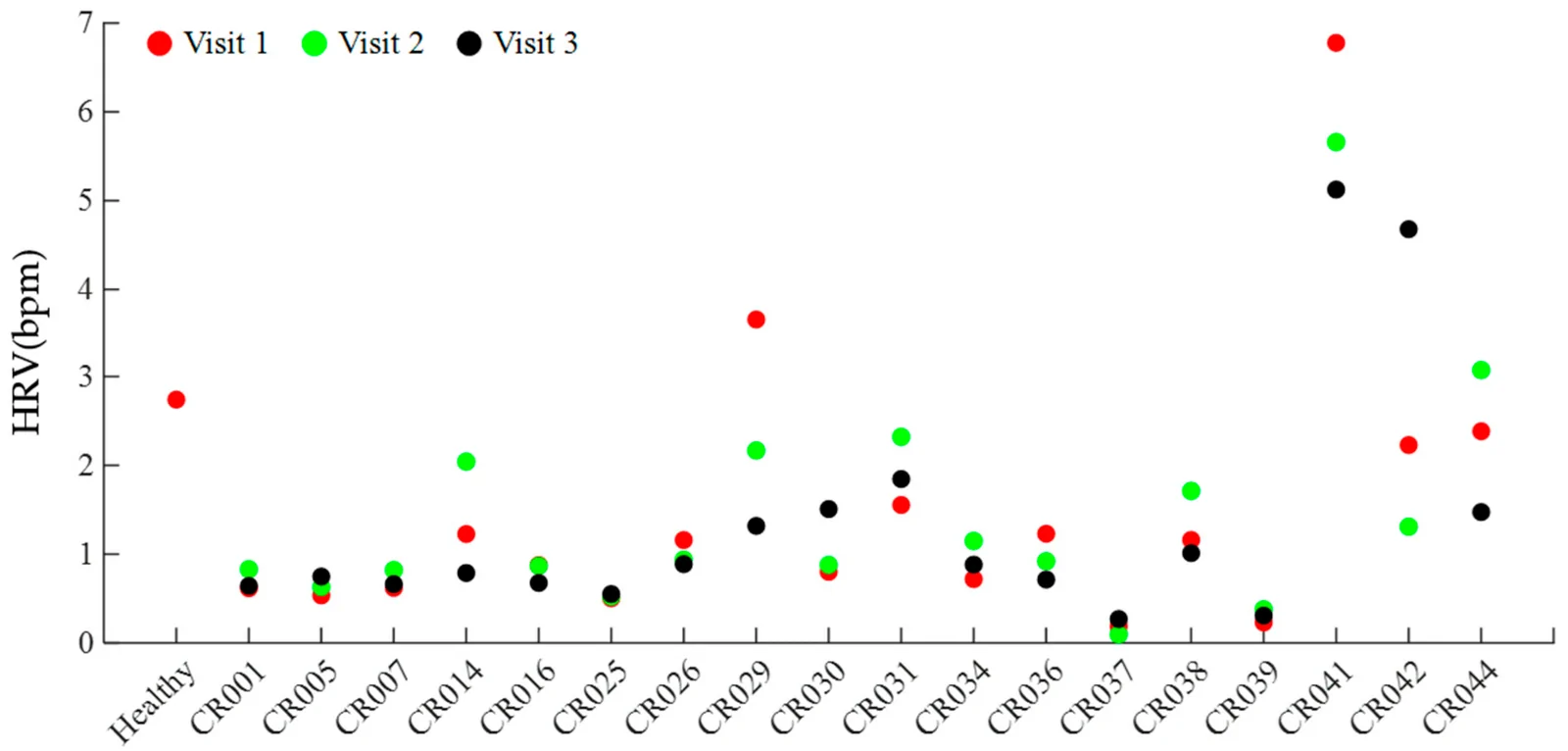
At-rest HRV of the subjects.

As shown in Figure 3, compared with the healthy reference, most patients exhibited low at-rest HRV, with minimal longitudinal changes. Several patients demonstrated distinct patterns: CR041 (AF, PCI), the only patient diagnosed with AF, had the highest HRV, a well-established characteristic of AF, and showed a longitudinal decrease in HRV; CR042 (CABG) showed the largest longitudinal fluctuations; CR029 (atrial-paced) exhibited the most pronounced longitudinal decrease in HRV; and CR037 and CR039 (chronic HF) maintained the lowest HRV. The noticeable longitudinal HRV changes observed in some patients likely reflect physiological variation rather than measurement variability alone, whereas the minimal changes observed in subjects such as CR001, CR025, and CR037 suggest relatively stable at-rest HRV across visits.

**Figure 4:**
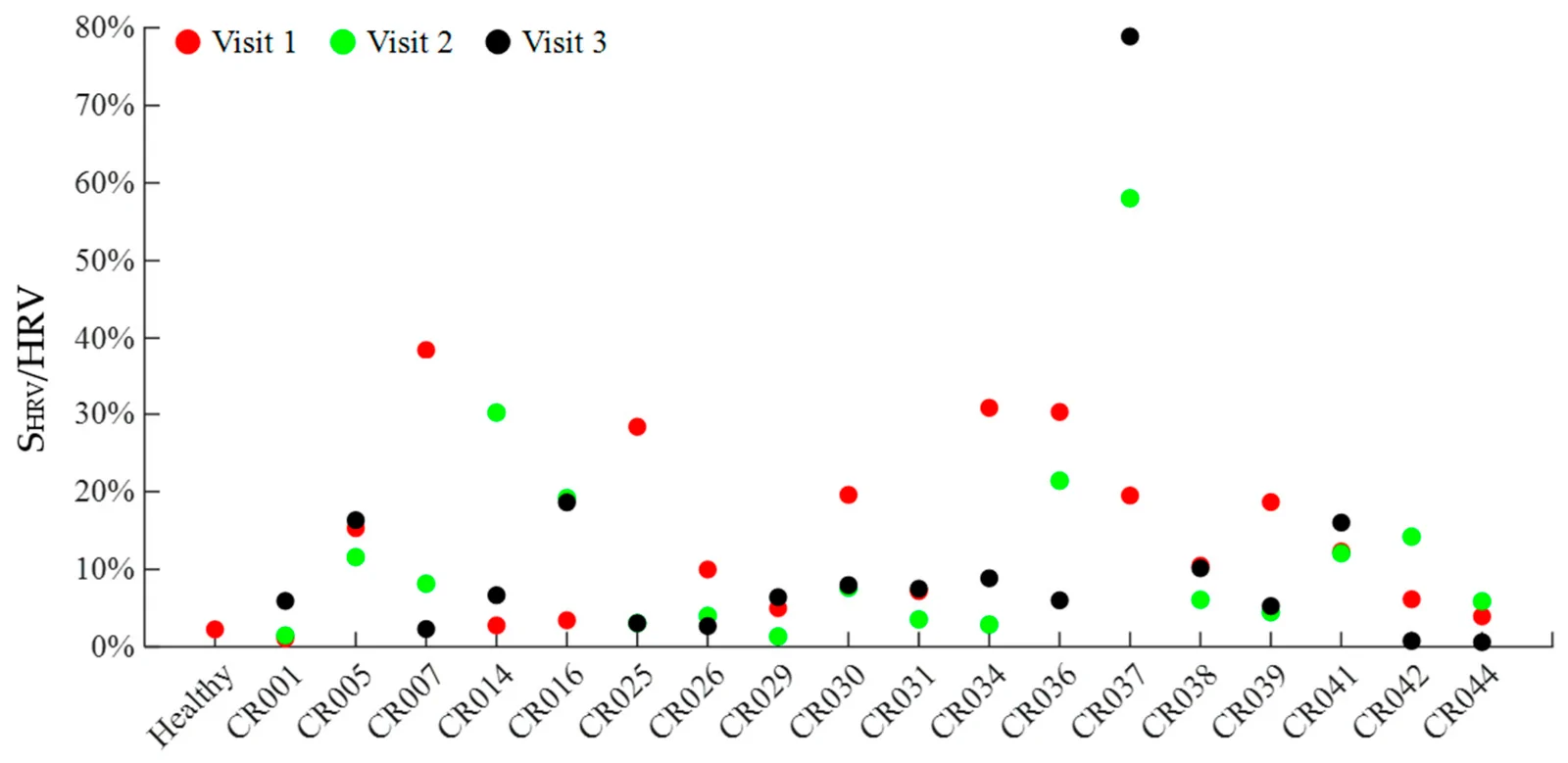
At-rest S_HRV_/HRV of the subjects.

As shown in Figure 4, the standard deviation of at-rest HRV across the first three harmonics, S_HRV_/HRV, was moderately elevated for most patients across visits as compared to the heathy reference. Some patients with elevated S_HRV_/HRV in Visit 1 showed reductions by Visit 3, approaching healthy levels. Notably, CR037 (chronic HF, CRT-D) showed an exceptionally large increase, and CR007 (NSTEMI) exhibited a notable decrease. At-rest S_HRV_/HRV was also elevated in CR041 (AF), consistent with a previous finding in AF based on finger PPG signals [24]. The noticeable longitudinal changes in S_HRV_/HRV observed in some patients likely reflect physiological variation rather than measurement variability alone, whereas the small changes observed in subjects such as CR001, CR005, and CR044 suggest relatively stable S_HRV_/HRV across visits.

**Figure 5:**
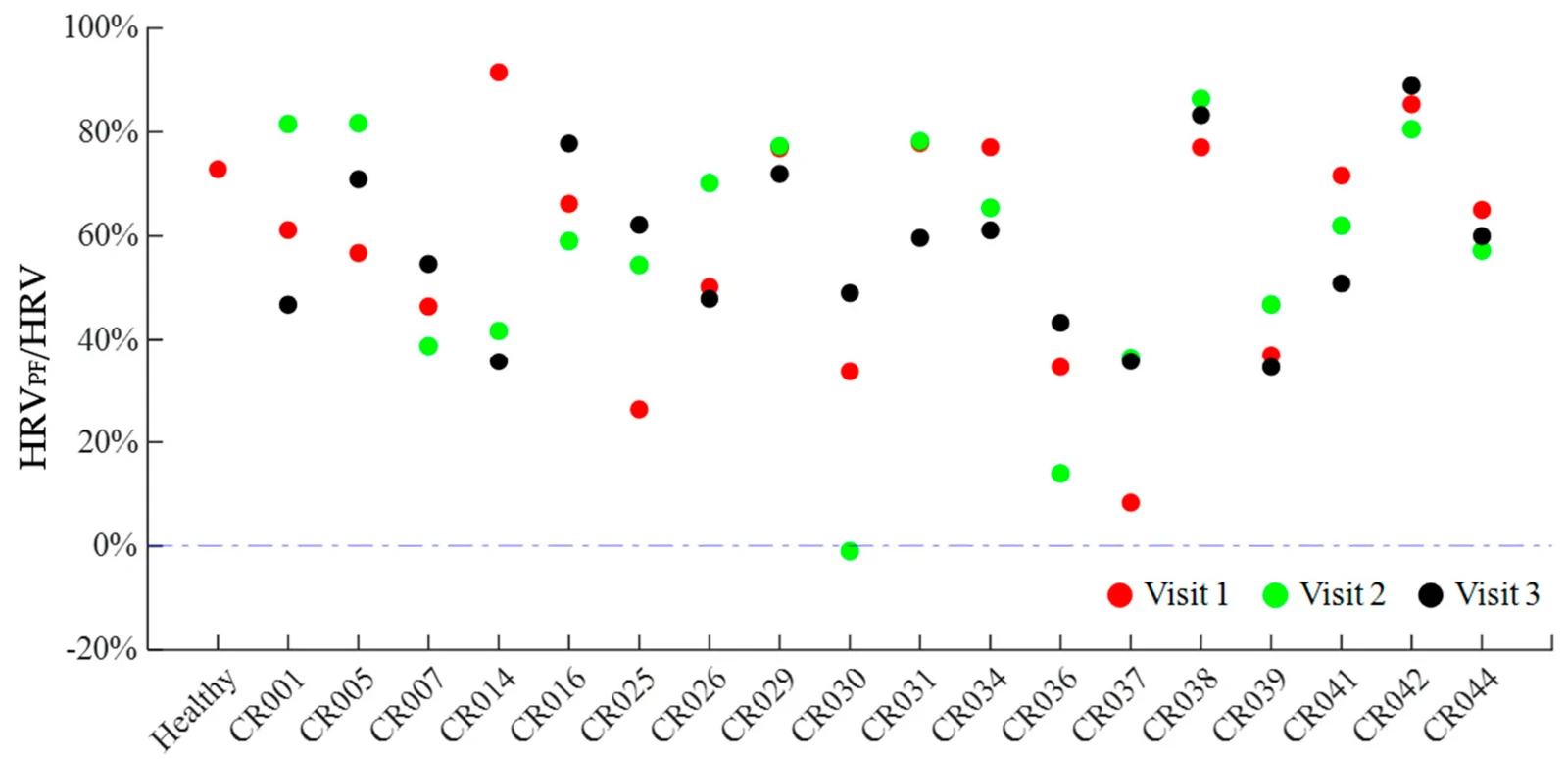
At-rest HRV_PF_/HRV of the subjects.

As shown in Figure 5, the contribution of other physiological factors (PFs), rather than respiration, to at-rest HRV and HRV_PF_/HRV was generally reduced. Notably, CR014 (CABGX6) showed the largest decrease from Visit 1 to Visit 3, whereas CR025 (MVR) and CR037 exhibited the largest increases. The noticeable longitudinal changes in HRV_PF_/HRV observed in some patients likely reflect physiological variation rather than measurement variability alone, whereas the small changes observed in subjects such as CR042 and CR044 suggest relatively stable HRV_PF_/HRV across visits. The negative HRV_PF_/HRV value for CR030 should be interpreted as a very small value rather than a physiologically meaningful negative contribution. It resulted from an error in the total HRV estimate derived from instantaneous frequency (IF) of a harmonic, which was slightly affected by MAs.

### 3.2. Respiration Parameters: RR and RM

**Figure 6:**
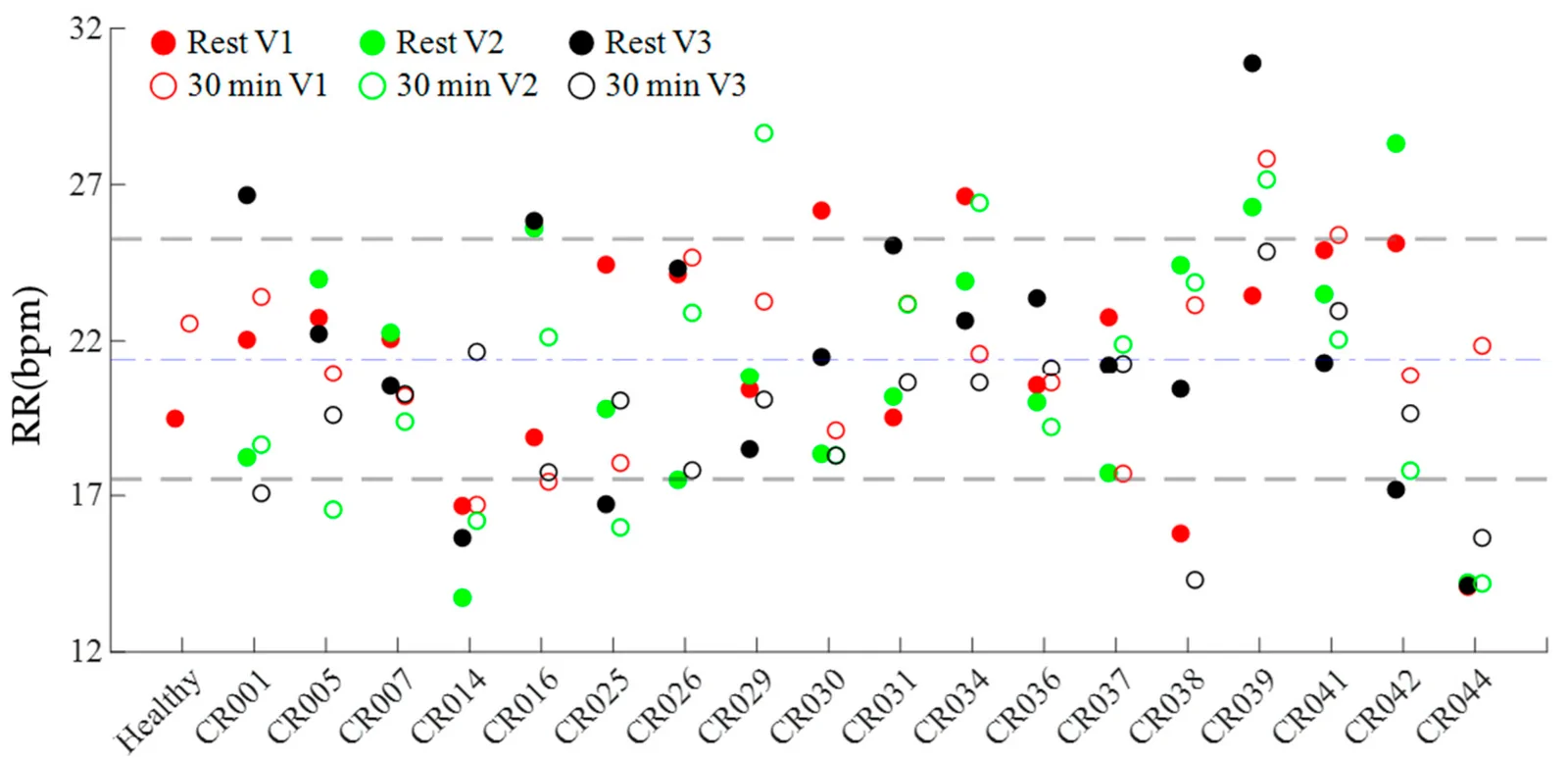
At-rest and 30 min post-exercise RR of the subjects (note: the dashed lines represent the mean ± standard deviation (SD) of the patient group, excluding the healthy reference).

As shown in Figure 6, at-rest RR was moderately elevated in most patients. Because the absolute magnitude of RR is relatively small, inter-visit differences can appear proportionally large. Distinct patterns were observed in several cases: CR039 (IPF) exhibited the highest RR, consistent with the subject’s underlying pulmonary pathology; CR025 (MVR) and CR042 (CABG) showed the largest longitudinal reductions; CR016 (chronic HF) demonstrated the largest increase; and CR014 (CABGX6) and CR044 (CABGX4) maintained the lowest RR.

Thirty-minute post-exercise RR remained slightly elevated in the healthy reference, increasing from 19 to 22 breaths/min, while patient responses were more variable. CR001, CR016, and CR039, all with elevated at-rest RR, exhibited marked reductions post-exercise. CR026 and CR038, despite normal at-rest RR, also showed large post-exercise decreases. In contrast, CR014, CR025, and CR042, all with low at-rest RR, displayed substantial post-exercise increases, whereas CR044, despite having the lowest at-rest RR, showed only a moderate elevation. These observations suggest that 30 min post-exercise RR responses vary with baseline RR and may also be influenced by underlying CV or pulmonary conditions.

Given the technical foundation of the SDOF-TF method and the acceptable quality of the recovered pulse-induced signals, the observed large longitudinal differences in at-rest RR and 30-min post-exercise RR across visits for individual subjects likely reflect physiological variation rather than measurement errors alone. However, because RR values are relatively small, small longitudinal differences, such as CR005 and CR007, may be more susceptible to measurement errors and should therefore be interpreted with caution.

**Figure 7:**
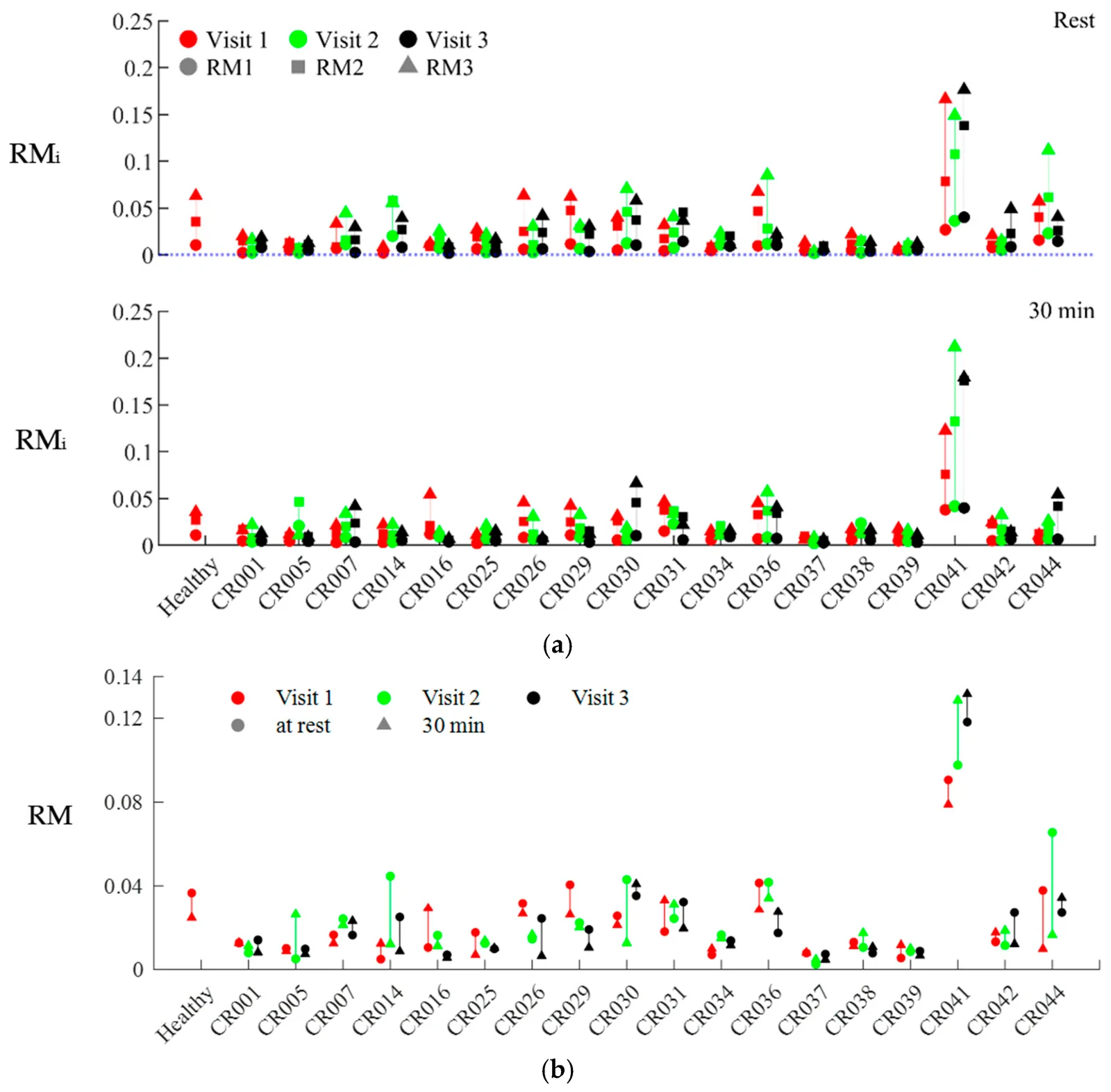
At-rest and 30 min post-exercise RM of the subjects. (**a**) RM of each harmonic. (**b**) Averaged RM across the three harmonics.

As shown in Figure 7a, the healthy reference exhibited an increasing trend in at-rest RM with harmonic order, with similar increments between consecutive harmonics. At-rest RM patterns varied across patients: while many retained an increasing trend with harmonic order, others exhibited weaker or disrupted trends, often accompanied by lower RM magnitudes. Among the patients, CR041 (AF) exhibited the highest at-rest RM values and maintained a consistent increase with harmonic order. Notably, CR041 was the only patient diagnosed with AF. The elevated RM observed in CR041 is consistent with findings from a previous study [24] that applied the SDOF-TF method to finger PPG signals for AF detection, in which AF subjects exhibited higher at-rest RM values than non-AF subjects. At the opposite extreme, several patients exhibited very low at-rest RM values, particularly during Visit 3, including CR005, CR016, CR034, CR037, CR038, and CR039.

Longitudinally, at-rest RM patterns varied across visits: some patients preserved an increasing trend with harmonic order, whereas others exhibited weak or disrupted trends. Several patients also showed noticeable changes in both RM magnitude and its harmonic-order pattern. For example, CR014 and CR042 showed a more pronounced increasing trend in RM with harmonic order, accompanied by increases in absolute RM values from Visit 1 to Visit 3. In contrast, CR036 exhibited a marked reduction in RM magnitude from Visit 1 to Visit 3, while preserving the increasing trend with harmonic order. CR031, CR034, and CR037, however, exhibited disrupted RM trends across harmonic orders in Visit 3.

For the healthy reference, RM at 30 min post-exercise generally preserved its increasing trend with harmonic order. Among the patients, some also maintained this trend, whereas others exhibited weakened or disrupted trends. To facilitate comparison between at-rest and post-exercise conditions, Figure 7b presents RM averaged across the three harmonics. Compared with the at-rest condition, RM decreased at 25 min post-exercise for the healthy reference, consistent with reports in the literature showing reduced respiration-induced modulation of HRV following exercise [21]. Similarly, CR029 (atrial-paced) exhibited consistent decreases in RM at 30 min post-exercise across all three visits. In contrast, the remaining patients showed increases or decreases in RM across visits, indicating heterogeneous post-exercise RM responses.

CR041 (AF) again exhibited a distinct pattern, with RM values at 30 min post-exercise remaining high relative to those of the healthy reference. Although RM decreased at 30 min post-exercise during Visit 1, it increased during Visits 2 and 3. Furthermore, during Visit 3, RM no longer exhibited the consistent increasing trend with harmonic order observed in the healthy reference. These findings demonstrate distinct RM behavior in CR041. Because rhythm-related HRV in AF can contribute to harmonic IF modulation and thus be captured by RM [24], the elevated RM values and their post-exercise changes may reflect a combination of respiratory modulation and AF-related rhythm variability, rather than respiratory modulation alone.

For subjects exhibiting small differences in at-rest RM across visits or small changes between at-rest and 30-min post-exercise RM in a visit, such as CR005 in Visits 1 and 3 and CR025 in Visits 2 and 3, it remains uncertain whether the observed difference in at-rest RM and change between at-rest and 30-min post-exercise RM primarily reflect physiological changes or measurement variability. In contrast, moderate or large differences in at-rest RM across visits or moderate or large differences between at-rest and 30-min post-exercise RM, as well as pronounced inter-subject differences in RM, are more likely to reflect underlying physiological changes or differences. However, because the quantitative threshold distinguishing meaningful physiological variation from measurement variability has not yet been established for RM, these interpretations remain qualitative.

### 3.3. Normalized Harmonic Amplitudes and Normalized APW

#### 3.3.1. Subject-Specific Comparison of Individual Normalized Harmonic Amplitudes

**Figure 8:**
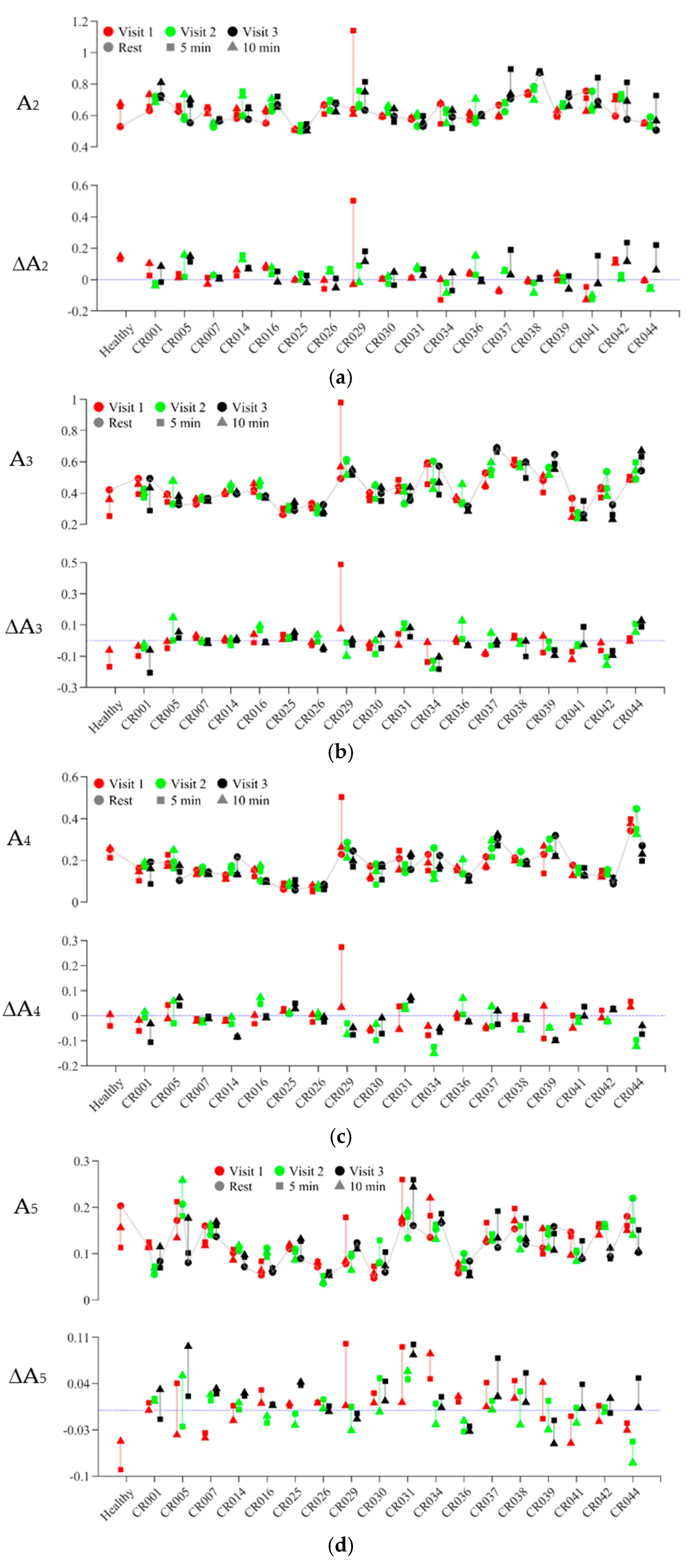

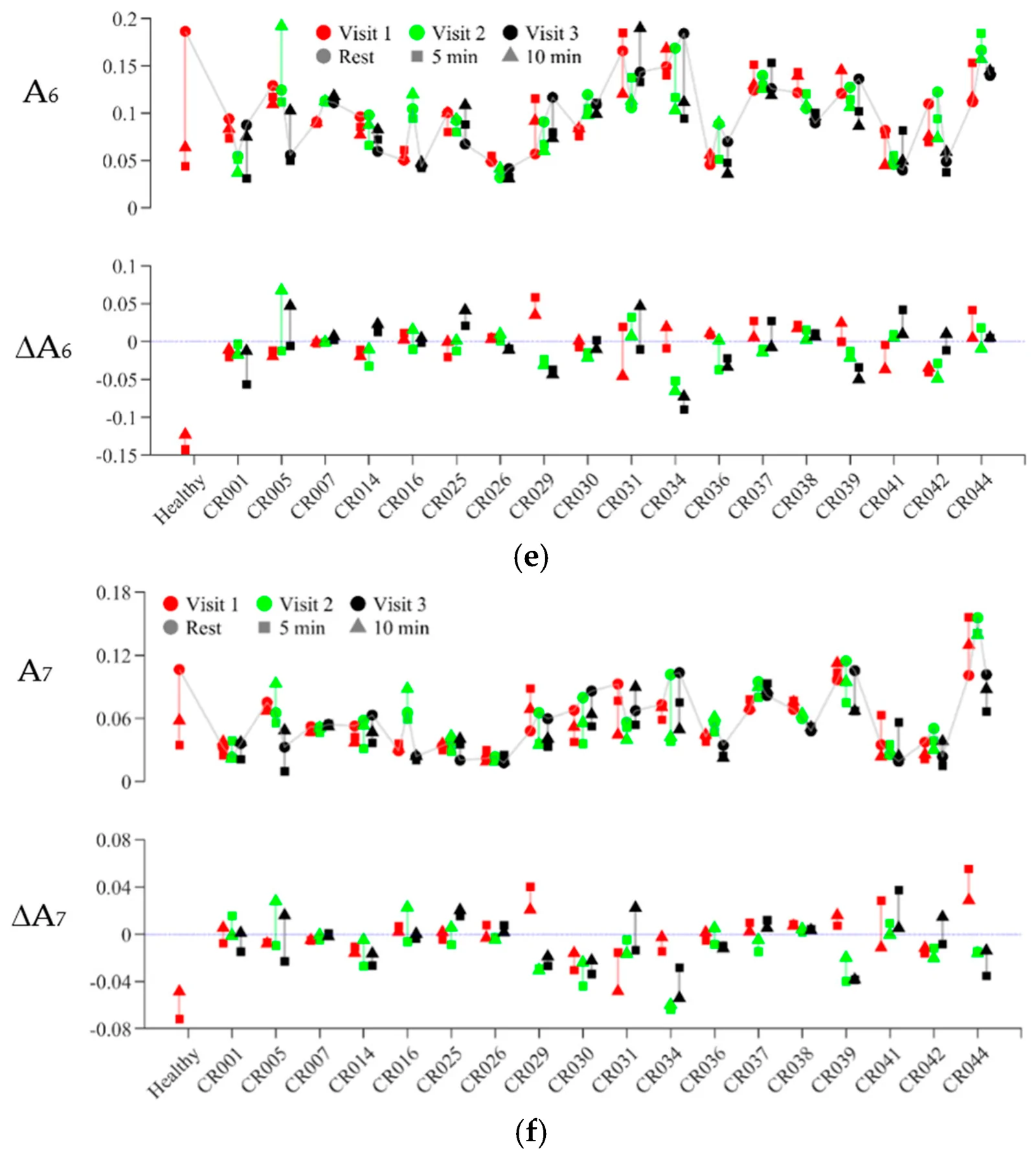
Normalized amplitudes of the higher harmonics (*i* > 1) and their changes in response to exercise: (**a**) 2nd harmonic; (**b**) 3rd harmonic; (**c**) 4th harmonic; (**d**) 5th harmonic; (**e**) 6th harmonic; (**f**) 7th harmonic (note: note that *A_i_* and *ΔA_i_* denote the *i*th normalized harmonic amplitude at-rest and the ith normalized harmonic change 5 min post-exercise, relative to at-rest).

Low arterial stiffness at rest has been associated with relatively large higher-harmonic amplitudes [45]. Figure 8 shows the at-rest normalized amplitudes, *A_i_*, of the first seven harmonics relative to the first harmonic. Comparisons of initial phases are omitted because of their high sensitivity to MAs and sensor noise [21,22].

Compared with the healthy reference, the patients generally exhibited slightly higher 2nd-harmonic amplitudes, comparable 3rd- and 4th-harmonic amplitudes, and markedly lower 5th- to 7th-harmonic amplitudes. The lower amplitudes of the higher-order harmonics are consistent with the reported association between increased arterial stiffness and greater attenuation of higher-order harmonics [45]. However, given the single healthy reference and the heterogeneity of the patient cohort, these differences are descriptive and do not independently establish increased arterial stiffness in patients.

Exercise modulated harmonic amplitudes in a systematic manner in the healthy reference: the 2nd harmonic increased notably, the 4th changed minimally, and the 3rd and 5th–7th harmonics decreased markedly. The largest deviations from baseline occurred at 5 min post-exercise, with partial recovery by 10 min. In comparison, patients generally exhibited a blunted response to exercise. Longitudinally, heterogeneous patterns were observed, with some patients showing more pronounced changes in specific harmonic amplitudes toward the healthy reference pattern, while others exhibited minimal change or changes in the opposite direction. These longitudinal interpretations should be made with caution, as variability in the TCS stack may introduce small fluctuations in normalized harmonic amplitudes. This consideration is particularly relevant to within-subject comparisons across visits and between measurement conditions (or time points within a visit), for which changes in TCS system parameters are expected to be relatively small, because slight variations may arise from differences in sensor placement, contact pressure, or tissue conditions. In contrast, direct inter-subject comparisons are less reliable because TCS system parameters may differ substantially among subjects. Accordingly, small changes within an individual across visits or between measurement conditions may reflect measurement variability, whereas moderate or large changes are more likely to reflect underlying physiological adaptations. However, because a quantitative threshold distinguishing physiological changes from measurement variability has not been established for normalized harmonic amplitudes, these interpretations remain qualitative.

CR029 displayed a particularly pronounced response. In Visit 1, this patient exhibited an exceptionally large increase in the second- to fourth-harmonic amplitudes at 5 min post-exercise, followed by a marked decrease between 5 and 10 min. CR029 was the only patient with atrial pacing. However, these pronounced changes were not observed in subsequent visits. Given the technical foundation of the SDOF-TF method and the acceptable quality of the recovered pulse-induced signals, the distinct pattern observed in Visit 1 likely reflects subject-specific physiological variation rather than measurement variability.

#### 3.3.2. Cross-Subject and Longitudinal Comparison of Normalized Harmonic Amplitude Profiles

In contrast to Section 3.3.1, which presents subject-level profiles of normalized harmonic amplitudes across all 18 patients, this section focuses on representative cross-subject comparisons and longitudinal patterns to improve visual clarity and interpretability. A subset of patients was selected to illustrate the variability in harmonic amplitude profiles across subjects, visits, and post-exercise time points. CR014 (CABGX6), CR034 (CABG), CR036 (CABGX5), CR044 (CABGX4), and CR037 (HF, CRT-D) were selected for cross-subject comparison, in addition to CR029 (atrial-paced) and CR041 (AF). CR029 and CR041 were also selected for longitudinal comparison to illustrate distinct response patterns.

**Figure 9:**
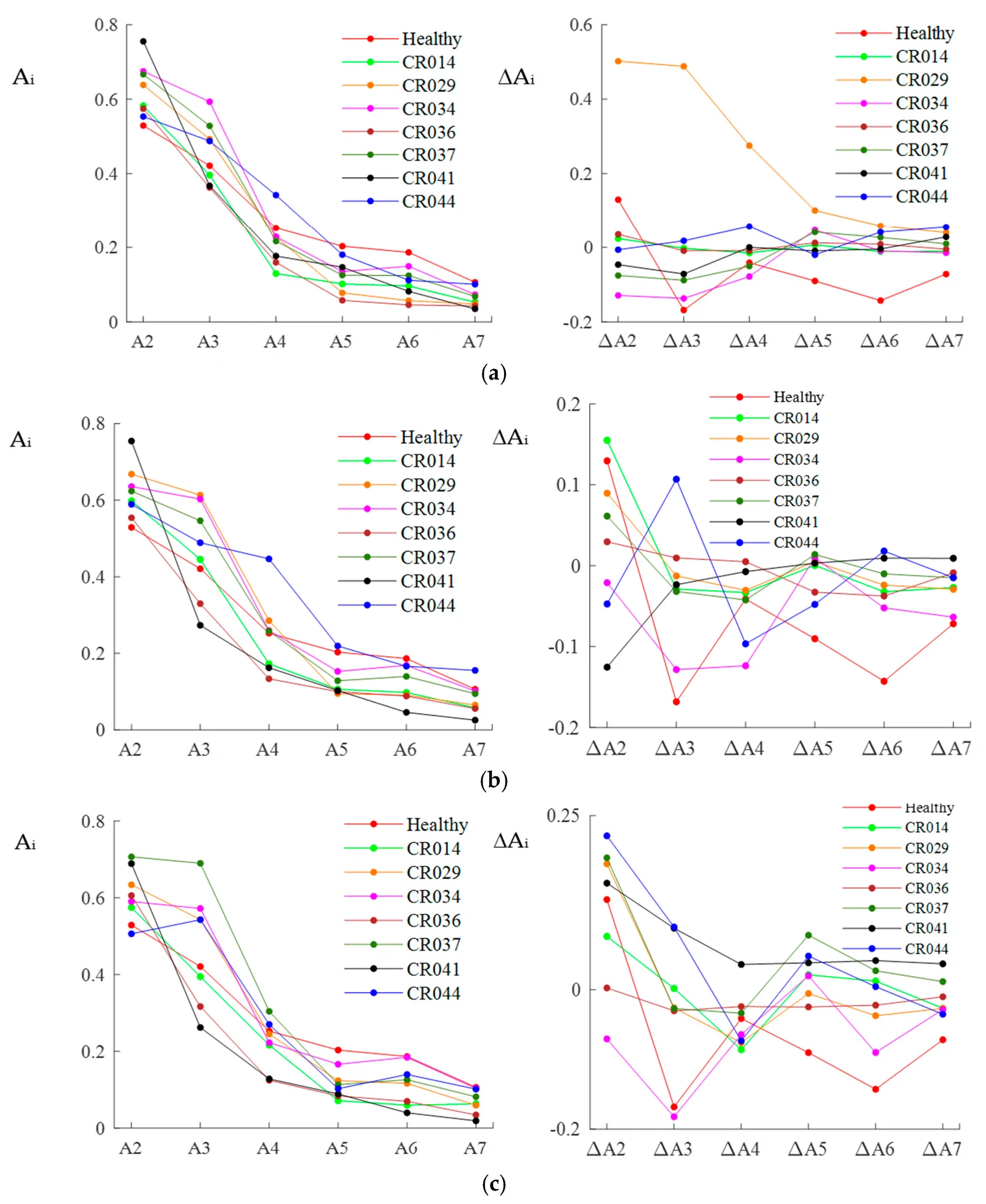
Cross-subject comparison of normalized harmonic amplitude profiling. (**a**) Visit 1. (**b**) Visit 2. (**c**) Visit 3 (note that *A_i_* and *ΔA*_i_ denote the *i*th normalized harmonic amplitude at rest and the *i*th normalized harmonic change 5 min post-exercise, relative to at-rest).

As shown in Figure 9, at-rest normalized harmonic amplitude profiles vary across patients and visits. Compared with the healthy reference, the 2nd harmonic amplitude is generally higher, whereas the higher-order harmonic amplitudes (5th–7th) are generally lower. The 3rd- and 4th-harmonic amplitudes show heterogeneous patterns across patients, with values both above and below those of the healthy reference. The exercise-related harmonic amplitude changes reveal more pronounced inter-subject differences within each visit. Specifically, these changes exhibit the following patterns across patients and visits: (1) the amplitude changes in the fifth to seventh harmonics remain consistently smaller than those of the healthy reference; (2) the 4th-harmonic amplitude changes are heterogeneous, with patient values both above and below the reference; (3) the 3rd-harmonic amplitude changes are generally smaller than those of the healthy reference, except for CR034 at Visit 3; and (4) longitudinally, an increasing number of patients exhibit 2nd-harmonic amplitude changes that exceed those of the healthy reference.

**Figure 10:**
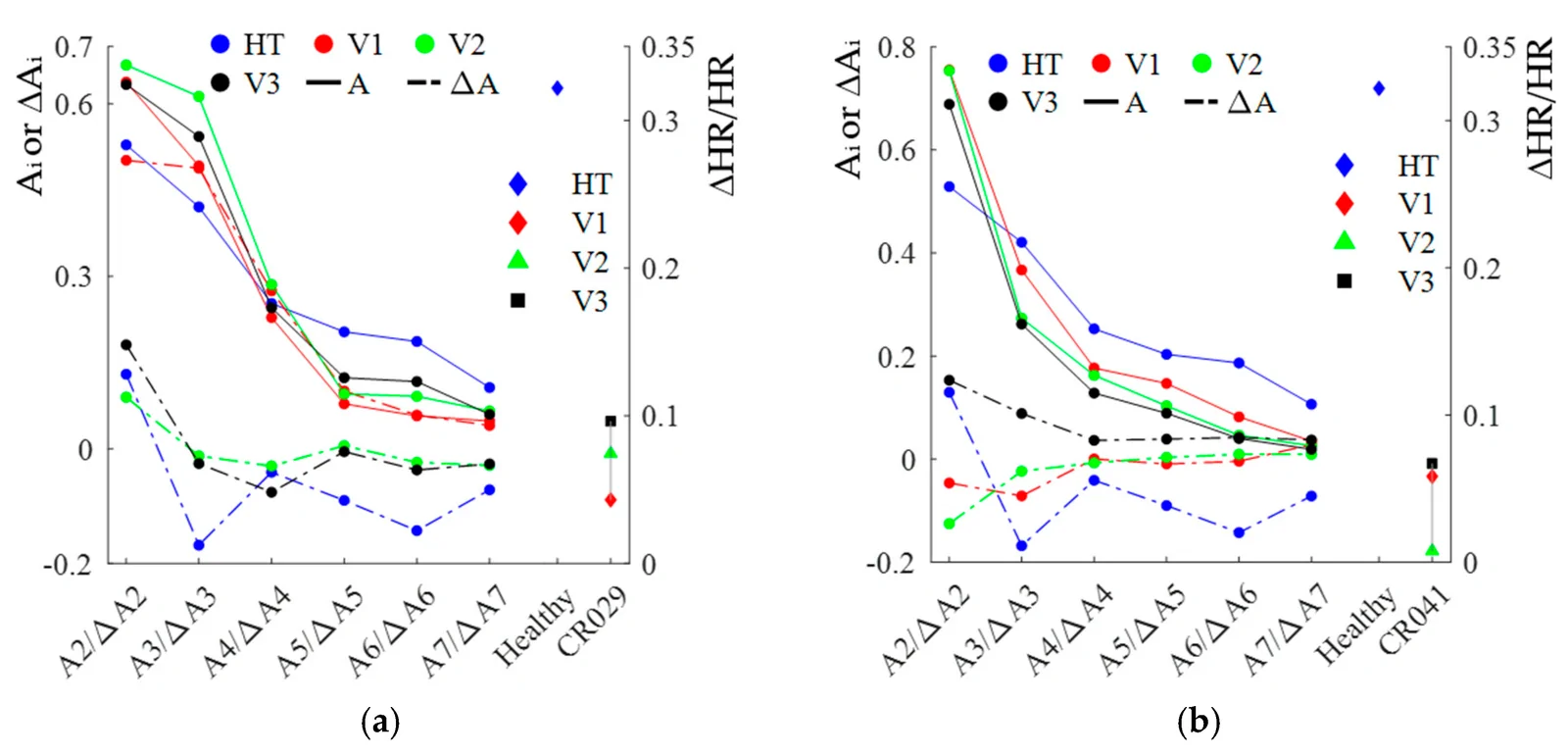
Longitudinal changes in normalized harmonic amplitude profiling. (**a**) CR029 (atrial-paced). (**b**) CR041 (AF). *A_i_* and *ΔA_i_* denote the *i*th normalized harmonic amplitude at rest and the ith normalized harmonic change 5 min post-exercise, relative to at-rest. The normalized heart rate change ΔHR/HR is also included. HT denotes the healthy subject; V1, V2, and V3 denote the first, second, and third visits, respectively.

As shown in Figure 10a, the 2nd- and 3rd-harmonic amplitudes both exhibit noticeable increases, whereas the 4th to 7th harmonic amplitudes show marked decreases in CR029 at-rest across visits, compared with the healthy reference. Although ΔHR/HR is lowest in Visit 1, the harmonic change profile is largest in that visit and differs substantially from those in the subsequent two visits. In contrast to the steady longitudinal increase observed in ΔHR/HR, the harmonic change profile does not exhibit a corresponding consistent longitudinal trend.

As shown in Figure 10b, only the 2nd harmonic amplitude increases while all the other harmonic amplitudes decrease in CR041 at rest across visits. Although ΔHR/HR is very close between Visits 1 and 3, the harmonic change profiling differ substantially. In particular, the harmonic change profile in Visit 3 is closer to that of the healthy reference.

Interestingly, despite the relatively similar at-rest harmonic amplitude profiles across visits, the harmonic change profiles reveal clear inter-visit differences. These observations suggest that exercise-related harmonic dynamics may capture variation that is not apparent from at-rest harmonic amplitude profiles alone. However, the present descriptive findings do not establish that these differences specifically reflect disease-related effects or autonomic regulation. There is no consistent correspondence between ΔHR/HR and the harmonic change profile.

#### 3.3.3. Normalized APW Between the RA and CA

To illustrate site-specific effects, a subset of representative subjects was selected: CR041 (AF) and CR029 (atrial-paced), representing two distinct rhythm conditions, together with CR001 and CR005 for comparison. Including all individual participants would be impractical, as it would amount to individualized interpretation of all 18 patients and is not suitable for paper presentation. Conversely, aggregated or averaged summaries may obscure important subject-specific physiological signatures.

**Figure 11:**
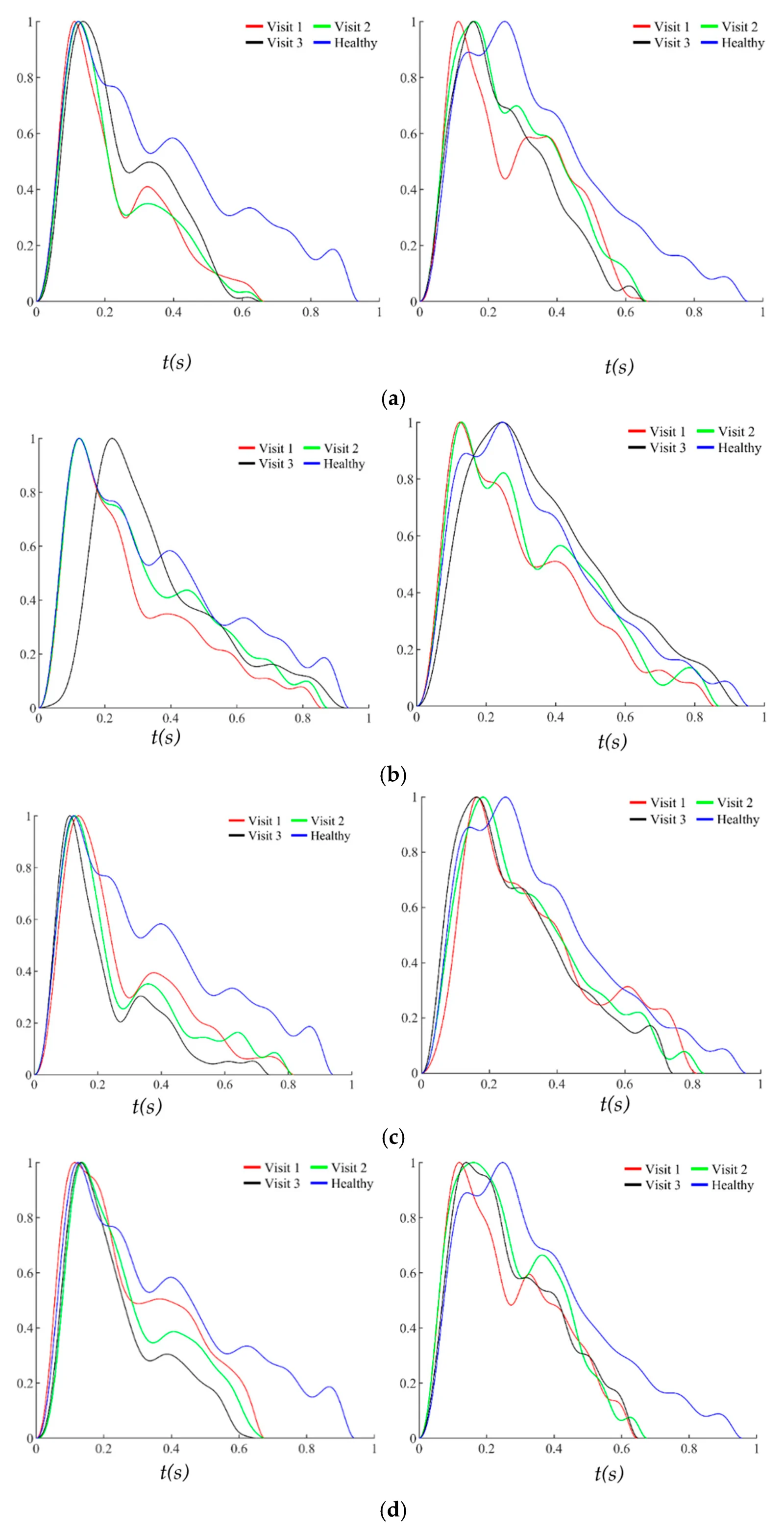
Comparison of normalized APW at rest between RA (left figure) and CA (right figure) across three visits of 5 subjects. (**a**) CR001. (**b**) CR005. (**c**) CR029 (atrial-paced). (**d**) CR041 (AF).

As shown in Figure 11, at the RA, the upstroke slope was similar between patients and the healthy reference, consistent with previous findings for CR039 [22]. In contrast, measurements at the CA showed more pronounced differences in upstroke slope between patients and the healthy reference, indicating that the observed site-specific differences in upstroke slope were more apparent at the CA than at the RA. Across all the subjects, HR measured at the RA and CA was comparable. Additionally, the DN was consistently located at a lower relative position in the RA waveform, consistent with known physiological differences between central and peripheral arterial sites.

For CR001, RA upstroke slope was comparable to the healthy reference, while CA upstroke slope was elevated. The DN followed the expected physiological pattern, with a lower relative position at the RA than at the CA.

For CR005, longitudinal changes were observed. Although at-rest HR decreased over time, the relative change (ΔHR/HR) increased. Upstroke slope at the RA remained similar to that of the healthy reference, while upstroke slope at the CA was higher during the first two visits. From visit 2 to visit 3, the change in resting HR was accompanied by noticeable changes in normalized APW and upstroke slope, suggesting that cardiac activity may modulate these parameters. A substantial change in normalized APW during visit 3 was observed simultaneously at both the RA and CA, indicating that this effect is unlikely measurement artifacts, but true physiological changes. The DN remained consistently lower at the RA, and HR remained similar between the two artery sites.

For CR005, longitudinal changes were observed. Although at-rest HR decreased over time, the relative change (ΔHR/HR) increased. Upstroke slope at the RA remained similar to that of the healthy reference, while upstroke slope at the CA was higher during the first two visits. From Visit 2 to Visit 3, changes in at-rest HR coincided with noticeable changes in normalized APW and upstroke slope, indicating that these parameters varied together over time without establishing a causal relationship. A substantial change in normalized APW during Visit 3 was observed simultaneously at both the RA and CA, suggesting a possible physiological change observed at both sites, although the contribution of measurement variability to the observed change remains unknown. The DN remained consistently lower at the RA, and HR remained similar between the two artery sites.

For CR029, at-rest HR in Visit 3 deviated more from the values in the first two visits; however, normalized APW did not show a corresponding pronounced change, unlike in CR005. This difference suggests that the longitudinal relationship between normalized APW and at-rest HR may vary across subjects. Upstroke slope in Visit 3 was similar to that of the healthy reference at the RA and only slightly elevated at the CA. The DN remained lower at the RA across all visits, and HR remained consistent between the two artery sites.

For CR041, upstroke slope at the RA was comparable to that of the healthy reference but consistently higher at the CA across all visits. HR remained similar between the two sites. Although the DN was higher at the RA than at the CA during Visit 1, it became lower at the RA than at the CA in subsequent visits, aligning with the expected physiological pattern.

#### 3.3.4. Features in Normalized APW at the RA and CA

By comparing the location of the DN at the RA in Figure 11 with the harmonic amplitudes in Figure 8, it appears that the 2nd and 3rd harmonics contribute substantially to the relative DN location in the APW. With increasing arterial stiffness, as observed in aging, lower-order harmonic amplitudes tend to increase, while higher-order harmonic amplitudes (5th to 7th) tend to decrease [45]. However, these higher harmonics are not explicitly visible in the APW. This suggests that the DN location at the RA may reflect stiffness-related changes in harmonic distribution, even though the contributions of the higher-order harmonics are not directly distinguishable in the waveform. In contrast, the upstroke slope at the RA remained comparable to that of the healthy reference in the patient group, suggesting that this feature showed limited variation relative to the reference at the peripheral site in the present cohort.

At the CA, however, the observed pattern differed: changes in upstroke slope were more pronounced, whereas differences in DN location were less evident. These findings suggest that the DN location at the RA and the upstroke slope at the CA may provide complementary, site-specific descriptions of APW morphology. Although these parameters may be influenced by arterial stiffness, they are not specific measures of arterial stiffness and may also vary with CV conditions and other confounding factors that affect APW morphology. Therefore, the radial DN location and carotid upstroke slope should be interpreted cautiously as potential indicators of stiffness-related changes, particularly given the inter-subject variability associated with the heterogeneous CV conditions and the limited sample size of the present cohort.

#### 3.3.5. Normalized APW Between the RA and CA of CR029 in Visit 1

**Figure 12:**
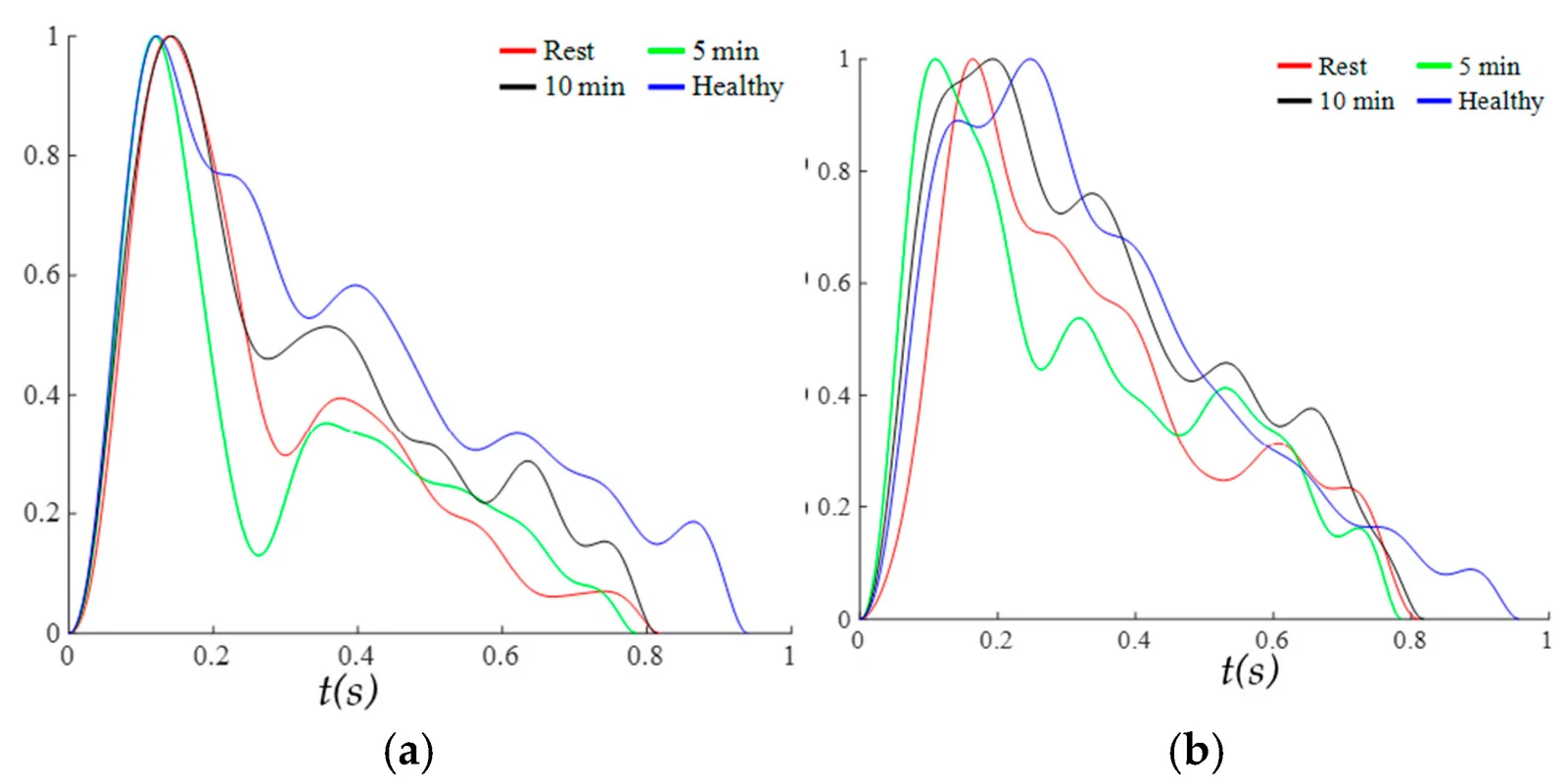
Normalized APW of CR029 at rest, 5 min and 10 min post-exercise in Visit 1. (**a**) RA. (**b**) CA.

Given the pronounced changes in normalized harmonic amplitudes during the post-exercise response in Visit 1 of CR029 (atrial-paced), the normalized APW at rest and 5 min and 10 min post-exercise is presented in Figure 12. The substantial increase in the 2nd–3rd harmonic amplitudes markedly altered the DN location at the RA.

At 5 min post-exercise, the upstroke slope at the RA increased compared with its values at rest and 10 min post-exercise, accompanied by an elevated HR—findings consistent with normal CV physiology. By 10 min post-exercise, both the upstroke slope and HR showed a trend toward their at-rest values. At the CA, a similarly pronounced shift in DN was observed at 5 min post-exercise, along with corresponding changes in upstroke slope and HR that were broadly consistent with expected post-exercise physiological responses. Notably, the increase in upstroke slope at the CA at 5 min post-exercise was substantially greater than that at the RA, suggesting a more pronounced response in the carotid waveform under these conditions.

Across all visits, HR measured at the RA and CA was comparable. Additionally, the DN was consistently located at a lower relative position in the RA waveform, consistent with the site-dependent differences in APW morphology observed in Figure 9. As illustrated by the upstroke slope differences between the RA and CA, the CA upstroke slope may be more responsive to within-subject physiological changes and may therefore warrant further investigation as a potential indicator of stiffness-related changes.

Overall, the co-occurring changes in APW morphology and HR in CR029 in Visit 1 are consistent with a physiological response to exercise. Given the technical foundation of the SDOF-TF method and the acceptable quality of the recovered pulse-induced signals, a physiological contribution is plausible; however, the contribution of measurement variability to the observed changes remains unknown. The DN shifted to an unusually low position in the RA waveform at 5 min post-exercise, warranting further investigation.

#### 3.3.6. Qualitative Evaluation of Normalized APW and Harmonic Amplitudes

As discussed in the previous study [21,22], the dependence of the normalized APW and harmonic amplitudes on the TCS stack limits the reliability of quantitative comparisons of APW-derived metrics across subjects or across arterial sites. However, qualitative comparisons—such as relative differences between subjects or longitudinal changes within the same subject at the same artery site—remain meaningful and comparatively reliable, as evidenced in Figure 12. Therefore, the interpretation of APW features in this study is primarily based on qualitative trends rather than absolute quantitative values.

As discussed in previous studies [21,22], the dependence of normalized APW morphology and harmonic amplitudes on the TCS stack limits the reliability of quantitative comparisons of waveform-based metrics across subjects and arterial sites. However, qualitative comparisons, such as longitudinal changes within the same subject at the same arterial site, may be more reliable because the TCS configuration is likely to vary to only a small extent, as evidenced in Figure 12. Therefore, the interpretation of APW features in this study is primarily based on qualitative trends rather than absolute quantitative values. Cross-subject and cross-site comparisons of waveform-based metrics are interpreted more cautiously because differences in TCS system parameters may contribute to the observed differences.

### 3.4. Summary of Observations

Overall, the results reveal both shared and heterogeneous patterns in CV dynamics across the 18 patients. In this study, “most” refers to the majority of the 18 patients, while acknowledging that a small subset may exhibit distinct or even opposing patterns due to individual physiological variability. As noted in Section 3.1 and Section 3.2, inter-visit differences were observed in the extracted CV parameters. Although the technical foundation of the SDOF-TF method and the acceptable quality of the measured pulse signals support the reliability of the extracted CV parameters, the relative contributions of physiological variation and measurement variability are difficult to distinguish when the observed changes are small. In contrast, moderate or large changes are more likely to primarily reflect physiological variation, although a contribution from measurement variability cannot be excluded.

First, HR and HRV analyses revealed heterogeneous patterns of HR dynamics and variability across patients relative to the healthy reference. While at-rest HR generally fell within the normal range, ΔHR/HR during post-exercise recovery was generally lower than that of the healthy reference, indicating smaller relative HR deviations during recovery. Except for relatively small inter-visit differences, changes in ΔHR/HR within individual patients are more likely to reflect physiological variation than measurement variability, although the latter cannot be entirely excluded. At-rest HRV was generally lower in patients than in the healthy reference, with relatively small longitudinal changes in many patients but pronounced visit-to-visit variations in a few. Notably, CR041, the only patient with AF, consistently exhibited high at-rest HRV (see Supplementary S2 in the Supplementary Document); HRV also remained elevated during post-exercise recovery, as evidenced by markedly fluctuating pulse-cycle intervals. At-rest S_HRV_/HRV also varied across patients and visits, with CR007 showing the largest longitudinal increase and CR037 the largest decrease. Compared with the healthy reference, at-rest HRV_PF_/HRV was lower in the patients. These findings describe differences in HR and HRV patterns but do not, by themselves, establish impaired autonomic regulation or attenuated CV function. The observed inter-subject and longitudinal variability highlights the potential value of individualized assessment, while physiological interpretation requires consideration of cardiac rhythm, medication effects, and other subject-specific factors.

Second, respiratory parameters, RR and RM, demonstrate substantial inter-subject variability. At-rest RR was elevated relative to the healthy reference in many patients and showed mixed post-exercise responses, which may reflect differences in baseline respiratory status, comorbid conditions, and other physiological or measurement-related factors. At-rest and 30-min post-exercise RM profiles showed heterogeneous patterns: while some patients preserved the increasing trend in RM with harmonic order observed in the healthy reference, others exhibited disrupted patterns and/or markedly reduced values. The only patient with AF (CR041) consistently showed elevated RM, consistent with the previous observation [24]. However, in AF, RM may also reflect irregular ventricular interval variability and should not be interpreted as a measure of respiratory modulation alone.

Third, harmonic analysis of normalized APW at the RA revealed differences between the patients and the healthy reference in the distribution of normalized harmonic amplitudes. Compared with the healthy reference, patients generally exhibited slightly elevated 2nd-harmonic amplitudes and markedly reduced higher-order harmonics (5th to 7th). These qualitative patterns are consistent with previously reported harmonic changes associated with age-related arterial stiffening [30] and may also reflect other individual physiological characteristics associated with CV conditions. However, arterial stiffness was not independently measured, and the healthy reference was a single individual who was not age-matched to the patients. Consequently, the observed harmonic differences cannot be attributed specifically to arterial stiffening, aging, or CV disease. Nevertheless, these patterns characterize the arterial pulse signals observed in this patient cohort and provide a basis for further investigation.

Fourth, comparison of normalized APW between the RA and CA in a subset of subjects revealed site-specific differences. At the RA, the upstroke slope showed relatively little variation from the healthy reference in the examined cases, whereas differences in harmonic amplitudes were more apparent. At the CA, upstroke-slope differences relative to the healthy reference were more pronounced in some subjects. Meanwhile, the location of the DN at the RA differed from that at the CA, with the DN typically appearing at a lower relative position in the RA waveform. Although MAs can complicate the extraction of HRV, RR, and RM at the CA, the normalized APW derived using the SDOF-TF method provided waveforms suitable for qualitative comparison in the analyzed segments. Across subjects, HR remained comparable between the two arterial sites. These observations suggest that CA and RA measurements provide complementary descriptions of APW morphology. However, the potential contributions of arterial stiffness, other physiological factors, and TCS-stack differences cannot be separated in this study. The anatomical proximity of the CA to the aorta may contribute to site-dependent waveform differences, but the present observations do not establish the CA as a more sensitive or specific measure of arterial stiffness.

Fifth, exercise was accompanied by changes in ΔHR/HR and harmonic amplitudes in patients, with peak deviations typically occurring at 5 min post-exercise, although in some patients the peak response shifted to 10 min and some responses had the opposite direction. Longitudinal trends remained heterogeneous, with the magnitude and direction of changes varying across patients and visits. However, no consistent correspondence was apparent between ΔHR/HR and harmonic-amplitude changes. Given the differences in patients’ physiological conditions and exercise intensity, the observed changes suggest some common trends but also substantial inter-subject variability in the magnitude, direction, and timing of post-exercise responses.

Sixth, the case study of CR029 in Visit 1 highlights the co-occurrence of pronounced changes in extracted parameters. Despite unusually large changes in harmonic amplitudes and DN location during post-exercise recovery, the concurrent changes in HR and APW morphology were broadly consistent with a post-exercise physiological response. Given the technical foundation of the SDOF-TF method and the acceptable quality of the recovered pulse-induced signals, a physiological contribution to these changes is plausible. These observations may indicate a change in arterial stiffness, but do not establish it. The contribution of measurement variability, particularly the TCS system parameters, cannot be excluded. The absence of similar changes in subsequent visits further underscores the subject-specific and visit-dependent nature of this response.

Seventh, compared with examining individual normalized harmonic amplitudes and their changes at 5 and 10 min post-exercise relative to at-rest, combining at-rest harmonic amplitude profiles with harmonic change profiles at 5 min post-exercise provides complementary information for characterizing subject-specific patterns across visits. These representations together help explain differences in normalized APW morphology between at-rest and 5 min post-exercise conditions. For instance, the downward shift in the DN at 5 min post-exercise relative to at-rest coincided with an increase in the 2nd harmonic amplitude. This correspondence illustrates how harmonic amplitude profiling can help interpret changes in waveform morphology.

Finally, longitudinal measurements were conducted three times over four weeks (week 1, week 2, and week 4) during the CR program. Analysis of pulse-derived CV parameters showed heterogeneous trends: some parameters increased, others remained relatively stable, and some decreased. These changes were observed while all patients were taking medication, which could have influenced the outcomes. The short time span further limits the detection of longer-term physiological adaptations. Given the heterogeneous cohort and descriptive nature of the study, these longitudinal changes should not be interpreted as evidence of clinical improvement or deterioration; rather, they characterize subject-specific patterns of CV variation over time as captured by tactile sensing and SDOF-TF-based analysis.

In summary, the combined analysis of HR, HRV, RR, RM, harmonic content, and APW morphology at rest and during post-exercise recovery demonstrated the feasibility of extracting and qualitatively examining multiple pulse-derived CV parameters in this heterogeneous patient cohort. The observed patterns varied substantially between patients and across visits, highlighting the value of individualized CV characterization while underscoring the need for further studies incorporating appropriate physiological reference measurements and larger cohorts to support physiological interpretation and establish clinical relevance.

## 4. Discussion

In this study, we investigated CV function across patients with diverse CV conditions using radial pulse waveform analysis, capturing HR, ΔHR/HR, HRV, standard deviation of HRV across the first three harmonics (S_HRV_/HRV), RM, RR, and normalized APW including harmonic amplitudes. The SDOF-TF method was employed to analyze the measured pulse signals, enabling high-resolution decomposition of harmonics and variability components over time. This approach provides insights into both autonomic regulation and vascular dynamics beyond conventional time-domain measures and allowed us to quantify CV dynamics at rest and during post-exercise recovery, revealing subject-specific CV patterns.

In this study, we investigated CV function across patients with diverse CV conditions using radial pulse waveform analysis, capturing HR, ΔHR/HR, HRV, standard deviation of HRV across the first three harmonics (S_HRV_/HRV), RM, RR, and normalized APW morphology, including harmonic amplitudes. While a micro-fabricated tactile sensor was used to acquire pulse signals at the RA and the CA, the SDOF-TF method was employed to analyze the measured pulse signals, enabling model-based decomposition of harmonic components and their temporal variations. The technical foundation of the SDOF-TF method, together with the acceptable quality of the recovered pulse-induced signals, supports the reliability of the extracted CV parameters. This approach enabled the qualitative examination of multiple pulse-derived CV parameters at rest and during post-exercise recovery, providing a basis for characterizing subject-specific patterns of physiological variation over time and exploring their potential physiological significance.

### 4.1. Common Features: At-Rest and Post-Exercise Response

Despite the heterogeneity of the cohort, several common patterns were observed. At rest, most patients exhibited HR within the normal range, whereas HRV was generally lower than that of the healthy reference. The relative HR change 5 min post-exercise (ΔHR/HR) was also generally lower than that of the healthy reference, indicating smaller relative deviations from at-rest HR. These observations describe differences in HR dynamics but do not, by themselves, establish impaired autonomic regulation.

Post-exercise responses in terms of APW morphology and harmonic amplitudes provided additional information on the temporal patterns of post-exercise responses. In some patients, the largest deviations in harmonic amplitudes and APW features occurred at 5 min post-exercise, followed by partial recovery at 10 min. In others, the peak deviation occurred at 10 min post-exercise, indicating variability in the timing of these responses. Compared with the healthy reference, however, these responses varied in magnitude and temporal pattern, rather than showing a uniform attenuation. In a few cases, HR at 5 min post-exercise was lower than at rest. These findings further illustrate the heterogeneity of post-exercise responses but do not, by themselves, establish impaired autonomic regulation.

Despite the variability in the magnitude and timing of these responses, the observed changes in HR and features associated with arterial stiffness (i.e., upstroke slope and higher-harmonic amplitudes) during recovery were broadly compatible with expected post-exercise physiological patterns. However, these observations do not establish changes in arterial stiffness or the integrity of the underlying regulatory mechanisms.

Harmonic analysis of normalized APW at-rest reveals a common qualitative pattern of harmonic-amplitude differences between the patients and the healthy reference that is compatible with previously reported changes associated with arterial stiffness. Specifically, elevated 2nd harmonic amplitudes combined with marked attenuation of higher-order harmonics (5th–7th) are generally observed at the RA. However, the 3rd and 4th harmonic amplitudes show heterogeneous behavior across patients, with values being either higher or lower than those of the healthy reference. These patterns indicate differences in the relative harmonic composition of the APW. The lower relative amplitudes of higher-order harmonics may reflect differences in the finer morphological features of the waveform, while the variability in the 3rd and 4th harmonics highlights inter-subject differences in waveform morphology at rest. Such differences may arise from multiple factors, potentially including ventricular–arterial interaction and wave transmission characteristics. However, because arterial stiffness and these mechanisms were not independently assessed, the observed harmonic differences cannot be attributed specifically to increased arterial stiffness or any single physiological mechanism.

At 5 min post-exercise, the observed redistribution of harmonic amplitudes—characterized by an increase in the 2nd harmonic, relative stability of the 4th harmonic, and attenuation of the 3rd harmonic and higher-order harmonics (5th–7th)—indicates a shift in the APW toward lower-frequency dominance and reduced harmonic richness. This pattern reflects a reduced contribution of higher-order harmonics associated with rapid waveform transitions and finer structural components of the APW, while the 2nd harmonic becomes more prominent in shaping the overall waveform contour. Physiologically, this adjustment is consistent with early post-exercise recovery, during which the CV system transitions from an exercise-driven state toward re-establishment of baseline hemodynamic conditions. In this phase, the pulsatile pressure waveform becomes less dominated by higher-frequency components associated with rapid cardiac ejection dynamics and more governed by global circulatory behavior, reflecting a temporary reorganization of CV control as the system restores resting-level interaction between ventricular stroke volume and the arterial response.

At 5 min post-exercise, the observed redistribution of harmonic amplitudes—characterized by an increase in the 2nd harmonic, relative stability of the 4th harmonic, and attenuation of the 3rd and higher-order harmonics (5th to 7th)—indicates a change in the relative harmonic composition of the APW, with greater prominence of the 2nd harmonic and lower relative amplitudes of several higher-order harmonics. This pattern alters the relative contributions of harmonic components to waveform morphology, potentially affecting both the overall waveform contour and finer morphological details. Physiologically, these changes occurred during early post-exercise recovery, when the CV system transitions from exercise toward resting conditions. The observed harmonic amplitude changes characterize subject-specific variations in arterial pulse dynamics during post-exercise recovery, reflecting changes in the underlying hemodynamic state. However, the regulatory mechanisms underlying these changes cannot be established from the present measurements alone, particularly given the heterogeneity of patients’ CV conditions and medication use.

These patterns are interpreted qualitatively, as absolute harmonic amplitudes depend on the TCS stack and are not directly comparable across subjects or arterial sites. The DN of APW at the RA is markedly lower than that of the healthy reference, compared with the CA. At the RA, the upstroke slope of the APW does not show meaningful differences between patients and the healthy reference, indicating limited sensitivity of this feature. In contrast, CA-based APW morphology reveals more pronounced differences in upstroke slope, suggesting greater sensitivity to stiffness-related alterations when assessed via upstroke slope.

These patterns are interpreted qualitatively, as absolute harmonic amplitudes depend on the TCS stack and are not directly comparable across subjects or arterial sites. The DN of APW at the RA is markedly lower than that of the healthy reference, whereas the difference at the CA is less pronounced. At the RA, the upstroke slope of the APW does not show meaningful differences between patients and the healthy reference, indicating limited discriminatory value in this comparison. In contrast, CA-based APW morphology reveals more pronounced differences in upstroke slope, suggesting that the CA upstroke slope may be more sensitive to subject-specific differences in waveform morphology. Although the upstroke slope of the CA waveform is commonly used as an indicator of arterial stiffness, whether the observed differences reflect changes in arterial stiffness requires further investigation.

Although MAs affect other parameters extracted at the CA, the normalized APW remains qualitatively more reliable after SDOF-TF processing than HRV, RR, and RM, which require fine time derivatives of the instantaneous frequency of the measured pulse signal. Accordingly, APW-related features are interpreted with an emphasis on relative patterns and longitudinal changes rather than absolute quantitative values. The harmonic amplitude distributions shown in Figure 8, Figure 9 and Figure 10 help explain the observed changes in normalized APW morphology in Figure 11 and Figure 12 across subjects and visits. It should be noted that, because the normalized APW depends on instantaneous phase values, which are more susceptible to TCS system parameters, qualitative features such as the upstroke slope and DN location may be less reliable than normalized harmonic amplitudes, even after MAs and sensor noise have been reduced through SDOF-TF processing. Therefore, fine-scale waveform details within the APW should be interpreted with caution.

RR and RM exhibit more variable behavior but still reflect common trends. Many patients show elevated at-rest RR and heterogeneous post-exercise responses, likely influenced by both CV and pulmonary factors. RM generally preserves its increasing trend with harmonic order, although its magnitude is often reduced. Together, these findings suggest a systemic reduction in physiological modulation across CV and respiratory domains. Finally, none of the patients exhibited a CV profile comparable to the healthy reference.

RR and RM exhibit more variable behavior but still reflect common trends. Many patients show elevated at-rest RR and heterogeneous post-exercise responses, potentially influenced by both CV and pulmonary factors. RM generally preserves its increasing trend with harmonic order, although its magnitude is often reduced. These findings characterize differences in respiratory-related and pulse-derived modulation patterns across patients, but do not establish a systemic reduction in physiological modulation across CV and respiratory domains. Finally, none of the patients exhibited a CV profile comparable to that of the healthy reference. This observation highlights differences in pulse-derived CV characteristics between the patients and the healthy reference, although the heterogeneity of the patient cohort precludes attributing these differences to a common underlying physiological mechanism.

### 4.2. Individualized CV Profiles

Beyond common trends, the results highlight substantial inter-subject and intra-subject variability, underscoring the importance of individualized CV profiling. Distinct subject-specific patterns emerge across multiple metrics, suggesting differences in individual CV responses and dynamics. The AF subject (CR041) consistently exhibits elevated HRV and RM, consistent with the substantial beat-to-beat variability associated with AF and potentially reflecting contributions from irregular ventricular rhythm to the extracted RM. In contrast, the chronic systolic HF patients (CR037 and CR001) demonstrate persistently reduced HRV and RM, indicating lower measured temporal variability in these indices but not necessarily establishing impaired autonomic or CV function. CR037 (CRT-D) shows the largest longitudinal increase in the magnitude of variation in SHRV/HRV, whereas CR001 (ICD) exhibits very low temporal variation in SHRV/HRV across visits. Despite similar HF classification, the two subjects also differ in at-rest HR, with CR001 presenting a relatively elevated at-rest HR (~90 bpm) and CR037 showing a lower at-rest HR (~60 bpm), illustrating the diversity of observed CV patterns among patients within the same disease category.

CR014 (male, 71–75 yr, CABGX6, beta-blocker use) and CR044 (male, 66–70 yr, CABGX4, no beta-blocker use) exhibited the lowest at-rest RR values in the cohort, yet both demonstrated pronounced longitudinal changes in at-rest HR and ΔHR/HR across visits. Their longitudinal trajectories differed: CR014 showed a substantial decrease in at-rest HR over time, whereas CR044 exhibited a marked increase. Despite these opposing trends in baseline HR, both patients displayed a clear longitudinal increase in ΔHR/HR, indicating progressively greater relative HR changes during post-exercise recovery compared with their respective at-rest values. Both patients had histories of extensive CABG, remained in SR, and had no significant arrhythmias documented during the study. These observations characterize distinct longitudinal patterns of pulse-derived CV parameters in two patients with similar surgical histories. However, the observed changes cannot be attributed specifically to physiological adaptation, recovery following revascularization, or changes in autonomic regulation or cardiac function, as other factors, including medication use and individual physiological variability, may have contributed.

In contrast, CR036 (female, 51–55 yr, CABGX5) did not exhibit similar longitudinal patterns despite a comparable surgical history. She maintained relatively stable at-rest HR and ΔHR/HR values across visits. Although age and sex may contribute to inter-individual differences in CV responses, their influence cannot be determined from these observations. This contrast highlights the heterogeneity of longitudinal CV patterns among patients with similar surgical interventions and supports the value of individualized monitoring to characterize patient-specific physiological variation over time.

The relationship between HR and APW morphology also varies between individuals. In CR005, longitudinal changes in HR are accompanied by corresponding changes in normalized APW, suggesting an association between changes in cardiac rate and APW morphology. However, this relationship is not universal; for instance, CR029 shows relatively large HR variation without corresponding changes in normalized APW, indicating subject-specific differences in the relationship between HR and waveform morphology. These observations further highlight that relationships among pulse-derived CV parameters are subject-specific and should be interpreted in the context of individual longitudinal patterns rather than as universal relationships, particularly because of inter-individual physiological variability.

The comparison between RA and CA measurements further supports the value of site-specific assessment. While RA-derived upstroke slope often appears similar between patients and the healthy reference, CA measurements reveal more pronounced differences in upstroke slope, potentially suggesting its higher sensitivity to physiological changes. Meanwhile, the DN in APW at the RA remains informative, often more revealing than at the CA. These findings suggest that the relative value of individual APW morphological features may depend on the measurement site. However, their sensitivity to CV disease or their utility for early detection cannot be established from the present descriptive comparisons alone.

The case of CR029 in Visit 1 illustrates the complexity of individualized responses. This subject exhibits pronounced, transient changes in harmonic amplitudes and APW morphology during post-exercise recovery, potentially associated with the subject’s atrial pacing and underlying cardiac condition, although these contributions cannot be established from the present measurements. Despite these atypical harmonic-amplitude changes, the changes in upstroke slope and DN location between at-rest and 5 and 10 min post-exercise were broadly consistent with expected variations in APW morphology during post-exercise recovery. However, the absence of a corresponding relationship between ΔHR/HR and normalized APW changes highlights the fact that different pulse-derived CV parameters may exhibit distinct response patterns. These observations underscore the importance of interpreting multiple parameters jointly rather than in isolation.

The effects of the outpatient CR program on the subjects were heterogeneous. Compared with the healthy reference, RA-derived parameters exhibited increases, decreases, or relative stability, with patterns varying across CV parameters and subjects. However, these changes cannot be attributed solely to the program, as longitudinal trajectories differed between subjects and across extracted CV parameters. All patients were receiving medication, and the short four-week observation period limits the ability to characterize longer-term physiological adaptations. Because CR is a 12-week program, measurements at four weeks may reflect an early, incomplete stage of rehabilitation. In addition, the results may have been influenced by differences in the time interval between each patient’s preceding intervention and the initiation of rehabilitation. Future studies with longer follow-up, larger cohorts, and appropriately designed comparison cohorts are needed to better distinguish program-associated changes from potential medication effects, natural disease progression, and other sources of physiological variability.

### 4.3. HRV Calculated Based on Instantaneous HR and Instantaneous Initial Phase

Conventional HRV analysis [46,47,48,49] partitions RR interval variability in an ECG signal into frequency bands: very low frequency (VLF, <0.04 Hz), low frequency (LF, 0.04–0.15 Hz), and high frequency (HF, 0.15–0.4 Hz). In short-term recordings (~5 min), analysis generally focuses on LF and HF components because reliable estimation of VLF oscillations is difficult when recording durations are limited. HF power is commonly associated with respiration-related vagal modulation, whereas LF reflects a mixture of autonomic influences and other physiological processes. Power spectral density (PSD) analysis, typically performed using fast Fourier transform (FFT)- or autoregressive (AR)-based methods, generally assumes approximate stationarity and often requires interpolation of unevenly sampled RR intervals to obtain an evenly sampled time series. These processing choices can influence the estimation and interpretation of HRV components, particularly at lower frequencies and in short recordings.

Conventional interpretations of CV autonomic regulation are primarily based on short-term HRV measures, where Root Mean Square of Successive Differences (RMSSDs) and HF power are used as proxies of vagally mediated respiration-related modulation [31,32,33,34]. Within this framework, CV disease is generally associated with reduced parasympathetic (vagal) activity and relatively increased sympathetic dominance.

In contrast, the present study defines HRV through instantaneous CV dynamics derived from the SDOF-TF framework [22]. Specifically, instantaneous HR captures the overall HRV. The respiration-induced HRV is further extracted using the instantaneous initial phase information, while the PF-induced HRV is obtained as the residual component after removing respiration-induced HRV. This decomposition provides a direct separation between respiration-driven modulation and other physiological sources of CV variability, rather than relying on frequency-band partitioning or beat-to-beat statistics. However, RM should not be interpreted as a specific index of RSA, because its physiological specificity has not been established. In particular, in AF, irregular rhythm-related variability may contain time-harmonic components that contribute to elevated RM, even in the absence of organized RSA.

Accordingly, the present findings should be interpreted in the context of established literature showing that CV disease is associated with reduced parasympathetic (vagal) activity and relatively increased sympathetic dominance. Conventional short-term HRV measures (e.g., RMSSD and HF power) primarily reflect respiration-related modulation (omitting VLF) and therefore typically decrease in disease states. In the present framework, the respiration-induced component is explicitly isolated, while the PF-induced component reflects the remaining CV variability beyond the respiration-induced component, including variability associated with VLF. Therefore, changes in PF-induced HRV should be interpreted as alterations in non-respiratory CV dynamics rather than a direct contradiction of established autonomic findings. This provides a complementary perspective by enabling separation of distinct components of CV variability, although their specific physiological origins cannot be inferred from this decomposition alone.

Nevertheless, the calculated IF-based HRV has demonstrated strong discriminatory ability, including 100% accuracy in AF detection [24], and clearly distinguishes the sole AF patient from the remaining subjects, supporting the ability of the proposed decomposition to retain sensitivity to abnormal CV dynamics, although this single-patient observation does not establish its generalizability.

### 4.4. Study Limitations

Several limitations should be considered when interpreting the findings of this study.

First, CA measurements are affected by substantial MAs, which limits their use primarily to qualitative analysis of APW morphology. Consequently, key parameters such as HR, HRV, RR, and RM are derived exclusively from RA measurements, which may restrict comprehensive cross-site comparisons of these parameters.

Second, the sample size is limited, and the cohort is heterogeneous with respect to underlying CV and pulmonary conditions. While this diversity enables the identification of subject-specific CV signatures, it limits the generalizability of the findings and precludes population-level statistical inference. Furthermore, differences in exercise intensity and individual physiological responses may contribute to the observed inter-subject variability, making it difficult to distinguish disease-related patterns from other sources of variation. Accordingly, the findings should be interpreted as descriptive observations rather than evidence of disease-specific patterns or diagnostic performance.

Third, the study includes a single healthy reference. Although the observed CV parameter patterns for this subject are consistent with established physiological findings reported in the literature [42], this does not capture the full range of normal inter-individual variability. Importantly, because arterial pulse measurements depend on the TCS stack, meaningful comparisons require a reference measured using the same sensing configuration. Therefore, the healthy subject serves as a device-specific baseline rather than a population-level normative reference. Future studies incorporating a larger healthy cohort measured under identical conditions are needed to strengthen comparative analysis.

Fourth, the interpretation of normalized APW and harmonic amplitudes is primarily qualitative. This is due to the influence of the TCS stack, which affects the quantitative values of waveform-derived measures and limits their direct comparison across subjects or arterial sites without matched sensing configurations. While qualitative trends and within-subject longitudinal changes remain meaningful, the lack of robust quantitative indices constrains the ability to establish standardized clinical metrics.

Fifth, the longitudinal assessment was conducted over a relatively short duration (four weeks across three visits), during which all patients were under ongoing medical treatment. The combined effects of medication, outpatient cardiac rehabilitation, lifestyle changes, and natural disease progression cannot be fully separated, and the short observation window may limit the detection of sustained physiological adaptations. In addition, the current protocol includes at-rest and extended post-exercise monitoring up to 1 hour, which may be unnecessarily long for the initial characterization of patient-specific CV signatures based on the present findings. The results indicate that patient-specific CV signatures can already be characterized using measurements at rest and 5 min and 10 min post-exercise. Importantly, from a practical standpoint, prolonged post-exercise monitoring may discourage patient participation and reduce recruitment feasibility, as observed in this study. These findings suggest that future protocols could prioritize at-rest and early post-exercise measurements, while retaining longer monitoring periods when needed to investigate recovery dynamics or other time-dependent physiological changes.

Sixth, although the study did not include quantitative error analysis or formal repeatability testing, the adopted methodology provides a technical basis for the extraction of CV parameters. The tactile sensor facilitates arterial pulse measurement, signal quality is checked before subsequent analysis, and the SDOF-TF method is designed to recover pulse-induced signals from measured sensor data by accounting for MAs and sensor noise. These methodological safeguards support the technical credibility of the extracted parameters but do not independently establish their measurement accuracy, repeatability, or clinical validity.

Seventh, although efforts were made to suppress MAs and sensor noise, residual effects may still influence the accuracy of higher-order harmonic components and phase-dependent APW reconstruction, particularly at the CA. As a result, some observed variations may reflect a combination of physiological changes and measurement-related factors.

Eighth, although the SDOF-TF framework has demonstrated strong performance in prior studies—including AF detection from an independent PPG dataset [24] and physiologically interpretable results in representative clinical cases [22]—formal validation of the present sensing modality against established clinical gold standards was not performed in this study. Such validation remains an important direction for future work.

Ninth, although independent ECG and respiratory measurements were not available in this study for comparison, such reference measurements also carry measurement uncertainties and should not be regarded as error-free ground truth. Further comparative studies using appropriate physiological references are needed to evaluate agreement and characterize the remaining measurement uncertainties. The present study therefore focuses on the technical feasibility of recovering the pulse-induced signal and characterizing individualized CV patterns, rather than establishing the clinical validity of the extracted parameters.

Finally, beyond examining individual CV parameters and their longitudinal changes, the present study demonstrates the feasibility of simultaneously assessing multiple CV parameters to provide a more comprehensive characterization of CV dynamics. The proposed framework enables extraction of multiple physiologically related parameters within a unified formulation, allowing evaluation of both individual parameter behavior and their collective evolution across visits. The observed individualized variations across multiple CV parameters suggest that their inter-parameter relationships may also be patient-specific. However, because individual parameters vary substantially in magnitude, these relationships cannot be reliably characterized through direct visual inspection alone and require further quantitative investigation.

### 4.5. Additional Considerations and Future Work

The present findings indicate that RA pulse measurements combined with SDOF-TF-based analysis provide a comprehensive framework for characterizing individualized CV dynamics. However, future studies should include larger and more diverse cohorts to improve generalizability. Further development of MA suppression techniques at the CA site is also required to improve the usability of CA measurements and enable more comprehensive assessment across arterial sites.

PPG offers a complementary approach to tactile pulse sensing but involves different transduction mechanisms and sensor–tissue interactions. In our previous PPG-based AF study [24], only the first three harmonics could be reliably extracted from finger PPG signals in the investigated critically ill-patient dataset, highlighting potential challenges in higher-order harmonic analysis. Both the SDOF-TF method and the TCS model used in this work can be applied to PPG signals; however, optical transduction and its associated effects on measured pulse signals are not explicitly accounted for in the present model. Future studies could compare finger PPG with RA tactile sensing to investigate their complementary capabilities and limitations in harmonic analysis and the extraction of time-based CV indices, while accounting for differences in measurement site and transduction mechanism.

Machine learning approaches may complement the proposed framework by facilitating automated characterization and interpretation of individualized CV signatures. Notably, implementation of the SDOF-TF algorithm for pulse signal analysis is computationally efficient and straightforward. The primary challenge lies not in the signal processing required to extract CV parameters, but in interpreting their longitudinal changes within individuals and comparing patterns across subjects, particularly when comparing at-rest and post-exercise states. This challenge arises from the substantial inter- and intra-subject variability in CV profiles and the potentially complex relationships among multiple extracted parameters. Future work should therefore prioritize systematic characterization of these relationships and development of interpretable approaches for identifying clinically meaningful patterns.

Overall, the long-term value of this work lies in its potential to support patient-specific CV assessment and inform more personalized approaches to CV monitoring and rehabilitation. In particular, the simplicity of both the tactile sensor-based pulse measurement and the proposed SDOF-TF framework offers the potential for rapid, on-site assessment of CV responses with relatively low computational burden. Such capability may facilitate longitudinal monitoring of physiological responses associated with medication and CR. Before these applications can support clinical decision-making, however, further studies must establish the measurement validity, interpretability, and clinical relevance of changes in the extracted CV parameters. Ultimately, this approach may support more individualized patient management by enabling CV dynamics to be assessed repeatedly under at-rest and post-exercise conditions with minimum burden on individuals and caregivers.

## 5. Conclusions

This study demonstrates the feasibility of applying the SDOF-TF method to RA pulse signals for characterizing CV dynamics in a heterogeneous cohort of cardiac patients. The extracted parameters revealed substantial inter- and intra-subject variability, together with potentially meaningful patient-specific patterns in resting CV dynamics and post-exercise responses. Qualitative harmonic signatures and the relative location of the DN provided complementary information on APW morphology, while CA-based APW analysis showed greater sensitivity to changes in the upstroke slope than RA-based analysis, highlighting the value of site-specific assessment. These observations support further investigation of individualized CV profiling but do not establish clinical diagnostic validity or confirm specific underlying physiological mechanisms.

Despite the limitations associated with MAs, the small and heterogeneous cohort, and the use of a single healthy reference, this study illustrates the potential of combining tactile pulse measurements with SDOF-TF-based signal analysis to characterize multiple aspects of CV dynamics. The adopted methodology, which integrates tactile pulse measurement, signal-quality screening, and SDOF-TF-based signal retrieval, provides a technical basis for more robust extraction of time-based CV indices. Although the effects of the TCS stack on APW morphology are recognized, they are not removed by the present methodology and may influence the interpretation of waveform-related parameters. However, quantitative measurement accuracy and repeatability have not yet been established and require further validation.

Future studies should include larger and more diverse cohorts, improve MA suppression at the CA site, and validate extracted CV parameters against appropriate physiological reference measurements. Further investigation of longitudinal changes and inter-parameter relationships may support more interpretable individualized CV assessment. Machine learning may complement this physically grounded framework by automating pattern characterization, once the clinical relevance of the extracted parameters has been established.

## Figures and Tables

**Table 1:** Baseline demographic and clinical characteristics of the 18 cardiac patients and the healthy reference, including CV conditions, cardiac rhythms, beta-blocker use, and implanted devices.

Subject ID	Gender	Age	BMI	Reason for CR	Rhythm	Beta Blocker	Devices
Healthy	Male	41	19.9	--	--	--	--
CR-001	Female	66–70	29.8	HF	SR	Yes	ICD
CR-005	Male	46–50	36.3	PCI	SR	Yes	None
CR-007	Female	66–70	38.2	NSTEMI	SR	Yes	None
CR-014	Male	71–75	34.5	CABGX6	SR	Yes	None
CR-016	Female	56–60	24.1	HF	SR	Yes	None
CR-025	Female	51–55	25.9	MVR	SR	No	None
CR-026	Male	76–80	36.3	TAVR	SR	No	None
CR-029	Female	66–70	25.6	HF	Atrial-paced	Yes	ICD
CR-030	Female	66–70	29.6	MI	SR	Yes	None
CR-031	Male	51–55	35.7	PCI and MI	SR	Yes	None
CR-034	Male	66–70	38.3	CABG	SR	No	None
CR-036	Female	51–55	35.2	CABGX5	SR	Yes	None
CR-037	Male	66–70	37.8	HF	SR	Yes	CRT-D
CR-038	Male	61–65	30.2	PCI	SR	Yes	None
CR-039	Male	71–75	22.6	PCI	SR	No	None
CR-041	Male	76–80	37.6	PCI	AF	Yes	None
CR-042	Male	66–70	29	CABG	SR	Yes	None
CR-044	Male	66–70	30.8	CABGX4	SR	No	None

Note: 1. Age ranges are reported rather than exact ages to protect the identities and confidentiality of the subjects. 2. The list of all the medications prescribed to different individuals in the cohort: ACE inhibitors (ACEis), beta blockers, angiotensin II receptor blockers (ARBs), aldosterone antagonists, HMG-CoA reductase inhibitors (statins) allopurinol, diuretics (furosemide and bumetanide), angiotensin receptor–neprilysin inhibitors (ARNIs), and sodium–glucose cotransporter-2 (SGLT2) inhibitors. Only beta blocker use is reported in the table because of its effects on CV and autonomic regulation.

**Table 2:** At-rest HR and maximum HR during exercise in the 18 cardiac patients and the healthy reference, along with the corresponding absolute and normalized HR differences (note that the HRs of some patients measured by the medical instrument are missing). HR measurements at-rest from the medical instrument were unavailable for some patients.

Subject	Visit	At-Rest HR (bpm)		HR_max_ (bpm)	HR_max_ − HR_sensor_ (bpm)	(ΔHR_max_ − HR_sensor_)/HR_sensor_
		HR_medical_	HR_sensor_			
Healthy		--	63.72	106.75	43.03	68%
CR-001	Visit 1	87.00	90.51	120.00	29.49	33%
	Visit 2	82.33	90.97	113.00	22.03	24%
	Visit 3	89.00	91.40	125.00	33.60	37%
CR-005	Visit 1	68.67	69.97	83.00	13.03	19%
	Visit 2	68.33	68.69	91.00	22.31	32%
	Visit 3	65.33	64.73	92.00	27.27	42%
CR-007	Visit 1	84.00	84.76	110.00	25.24	30%
	Visit 2	75.67	74.81	114.00	39.19	52%
	Visit 3	76.33	75.97	114.00	38.03	50%
CR-014	Visit 1	73.67	74.61	96.00	21.39	29%
	Visit 2	69.00	68.38	107.00	38.62	56%
	Visit 3	54.33	53.39	95.00	41.61	**78%**
CR-016	Visit 1	77.00	77.52	86.00	8.48	11%
	Visit 2	85.33	86.01	108.00	21.99	26%
	Visit 3	87.67	89.38	106.00	16.62	19%
**CR-025**	Visit 1	75.67	77.95	104.00	26.05	33%
	Visit 2	78.33	80.40	107.00	26.60	33%
	Visit 3	87.67	89.92	109.00	19.08	21%
**CR-026**	Visit 1	81.67	84.39	96.00	11.61	14%
	Visit 2	82.67	83.91	103.00	19.09	23%
	Visit 3	85.00	86.55	104.00	17.45	20%
CR-029	Visit 1	73.00	73.25	93.00	19.75	27%
	Visit 2	71.00	73.62	106.00	32.38	44%
	Visit 3	81.33	81.03	115.00	33.97	42%
CR-030	Visit 3	73.33	70.76	122.00	51.24	**72%**
	Visit 2	65.67	68.02	106.00	37.98	56%
	Visit 3	60.67	61.69	106.00	44.31	**72%**
CR-031	Visit 1	68.67	66.96	108.00	41.04	61%
	Visit 2	67.33	66.36	112.00	45.64	**69%**
	Visit 3	68.67	69.79	119.00	49.21	**71%**
**CR-034**	Visit 1	76.33	77.32	97.00	19.68	25%
	Visit 2	73.33	72.87	98.00	25.13	34%
	Visit 3	73.00	75.78	107.00	31.22	41%
CR-036	Visit 1	64.33	66.02	92.00	25.98	39%
	Visit 2	61.33	63.47	88.00	24.53	39%
	Visit 3	73.00	74.64	103.00	28.36	38%
CR-037	Visit 1	59.67	60.02	88.00	27.98	47%
	Visit 2	59.67	59.99	86.00	26.01	43%
	Visit 3		60.02	88.00	27.98	47%
CR-038	Visit 1		72.88	100.00	27.12	37%
	Visit 2		71.38	103.00	31.62	44%
	Visit 3		78.27	109.00	30.73	39%
**CR-039**	Visit 1		71.28	95.00	23.72	33%
	Visit 2		72.20	103.00	30.80	43%
	Visit 3		71.33	110.00	38.67	54%
CR-041	Visit 1		87.86	140.00	52.14	59%
	Visit 2		88.46	160.00	71.54	**81%**
	Visit 3		89.48	150.00	60.52	**68%**
CR-042	Visit 1		84.93	118.00	33.07	39%
	Visit 2		80.57	122.00	41.43	51%
	Visit 3		86.93	144.00	57.07	66%
**CR-044**	Visit 1		62.80	86.00	23.20	37%
	Visit 2		55.28	95.00	39.72	**72%**
	Visit 3		70.03	103.00	32.97	47%

Note: the patients not taking beta blockers and the normalized HR differences exceeding that of the healthy reference are highlighted in bold.

## Data Availability

Due to institutional policy and applicable privacy regulations, individual-level patient data will not be shared with external investigators or repositories. These restrictions are necessary to protect patient confidentiality and comply with institutional governance of clinical data resources.

## Supplementary Materials

The following supporting information can be downloaded at https://www.mdpi.com/article/10.3390/diagnostics16213579/s1, Supplementary S1. Description of the exercise types performed by the study participants in the outpatient cardiac rehabilitation program. Supplementary S2. Measured arterial pulse signals at the RA and the CA of CR041 (AF patient) at-rest and during post-exercise recovery in Visit 1. Figure S1. At-rest. Figure S2. 5 min post-exercise. Figure S3. 10 min post-exercise. Figure S4. 20 min post-exercise. Figure S5. 30 min post-exercise. Figure S6. At-rest. Figure S7. 5min post-exercise. Figure S8. 10 min post-exercise. Figure S9. 20 min post-exercise. Figure S10. 30 min post-exercise.

## Abbreviations

The following abbreviations are used in this manuscript:

CRT-D	Biventricular Implantable Cardioverter Defibrillator
ICD	Implantable Cardioverter-Defibrillator
CABG	Coronary Artery Bypass Grafting
HF	Heart Failure
IPF	Idiopathic Pulmonary Fibrosis
MI	Myocardial Infarction
MVR	Mitral valve replacement
NSTEMI	Non-ST-segment Elevation myocardial Infarction
PCI	Percutaneous Coronary Intervention
SR	Sinus Rhythm
TAVR	Transcatheter Aortic Valve Replacement
AF	Atrial Fibrillation
CR	Cardiac rehabilitation
CV	Cardiovascular
APW	Arterial pulse waveform
TCS	Tissue-contact sensor
TVSPs	Time-varying system parameters
MA	Motion artifacts
DN	Dicrotic notch
SDOF-TF	Single-degree-of-freedom time–frequency
IF	Instantaneous frequency
BD	Baseline drift
HR	Heart rate
HRV	Heart rate variability
RSA	Respiratory sinus arrhythmia
RM	Respiration modulation
RR	Respiration rate
CA	Carotid artery
RA	Radial artery
