# Supplementary material for: Subject-Specific Cardiovascular Signatures Revealed by Arterial Pulse Signals in Patients with Heart Diseases—Part I: General Cases
Source: Diagnostics (Basel). 2026 Oct 29;16(21):3579. doi: 10.3390/diagnostics16213579 (PMC13761825; doi:10.3390/diagnostics16213579)
Supplement: Supplementary file 1 [file diagnostics-16-03579-s001.zip › diagnostics-4558565-supplementary.pdf]

Supplementary Document for

Subject-Specific Cardiovascular Signatures Revealed by Arterial Pulse Signals in Patients With Heart Diseases — Part I: General Cases

Supplementary S1

Description of the exercise types performed by the study participants in the outpatient cardiac rehabilitation program

- NuStep A&L (arms and legs)
- NuStep A (Arms only)
- NuStep L (legs only, seated stepper)
- Treadmill
- Bike Ergometer (upright bike)
- SCIFIT (recumbent bike - legs only)
- SCIFIT Arms (Arm ergometer only)
- Arm Ergometer
- Octane - Seated Elliptical
- Elliptical
- Rowing Machine
- weights (2-10lb weights)

Supplementary S2

Measured arterial pulse signals at the RA and the CA of CR041 (AF patient) at-rest and during post-exercise recovery in Visit 1

RA measurement

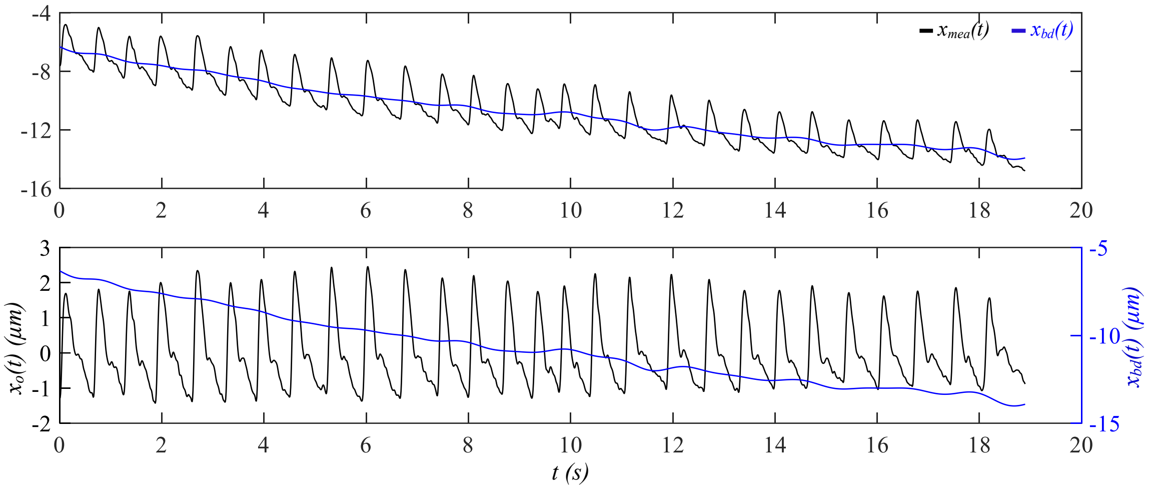

Figure S1. At-rest

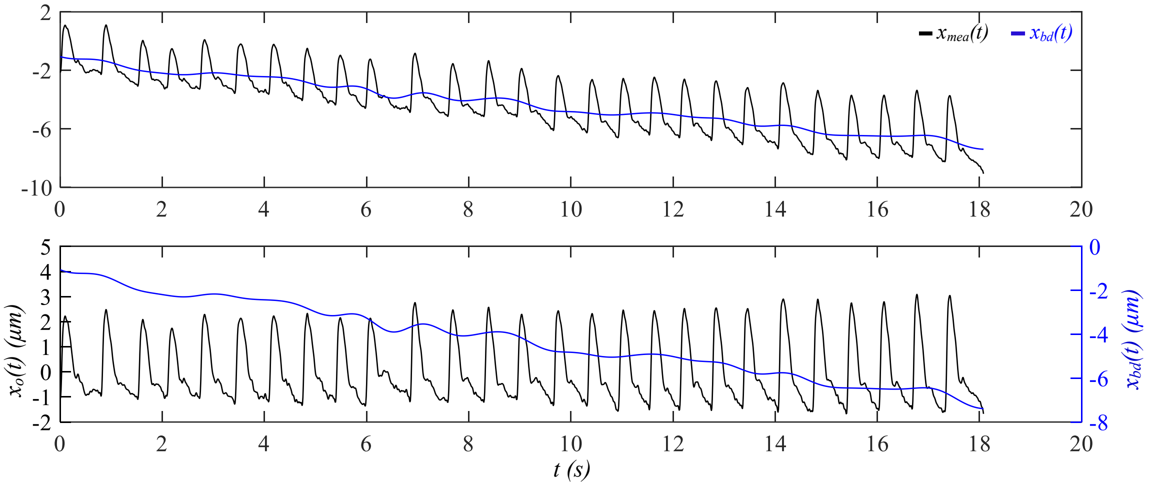

Figure S2. 5min post-exercise

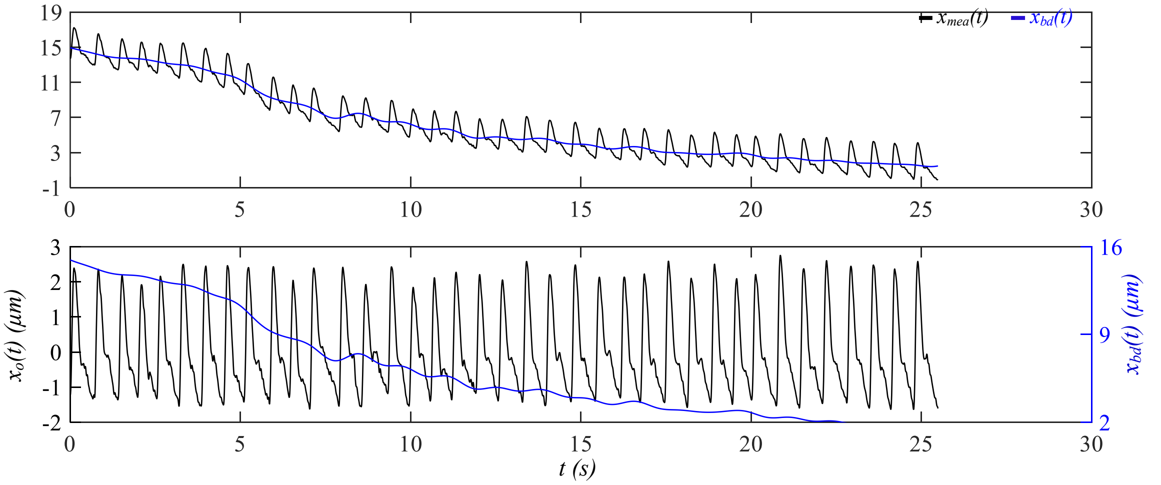

Figure S3. 10min post-exercise

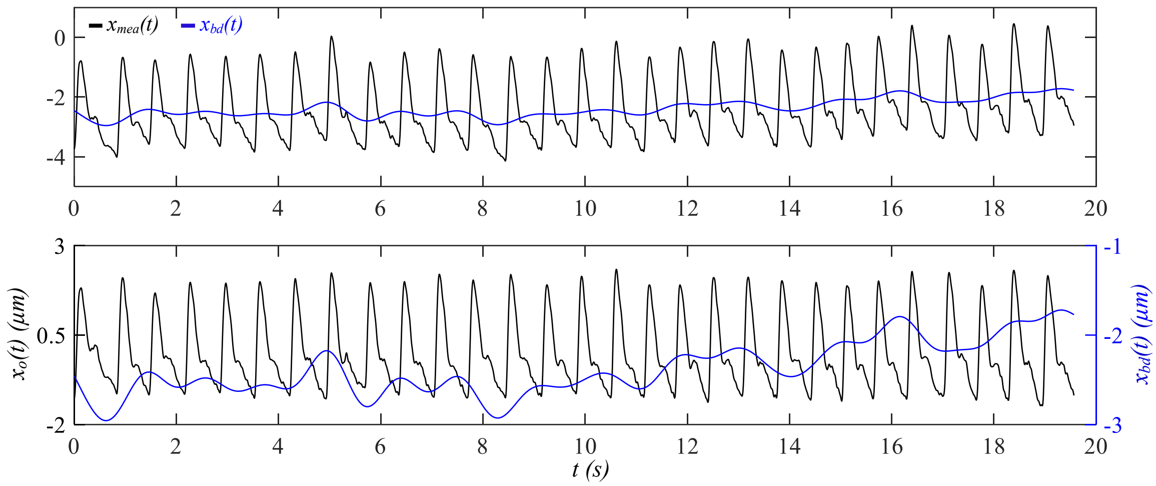

Figure S4. 20min post-exercise

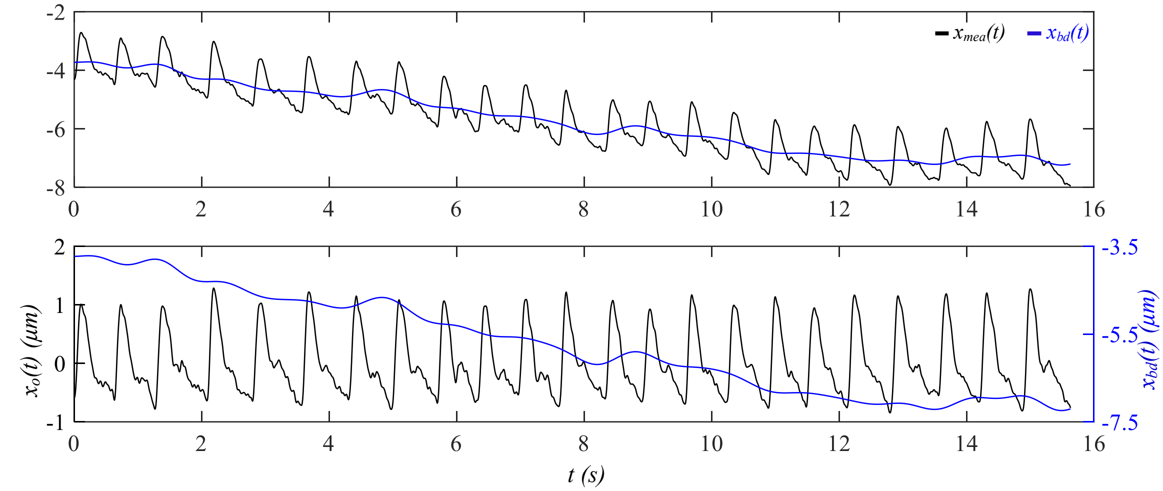

Figure S5. 30min post-exercise

CA measurement

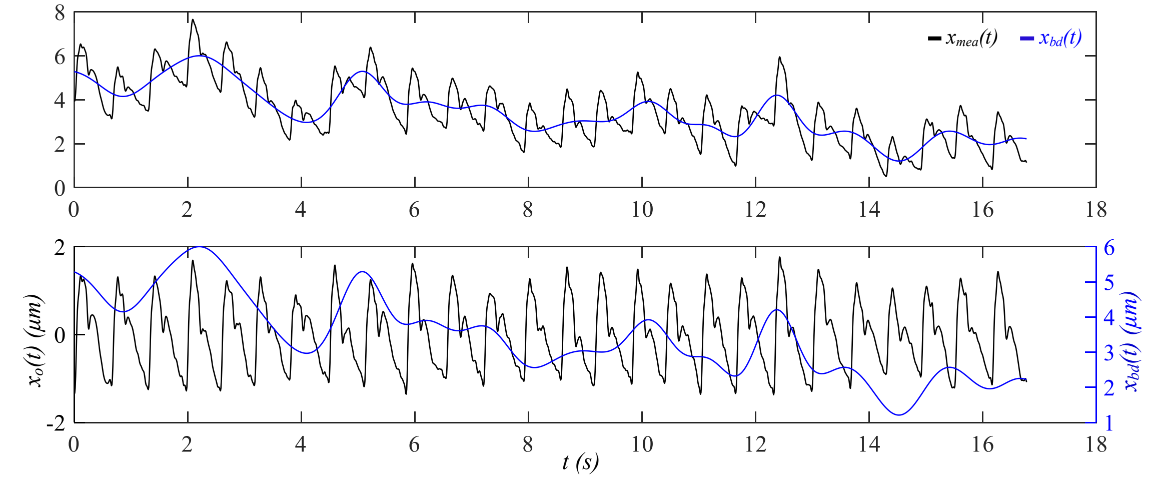

Figure S6. At-rest

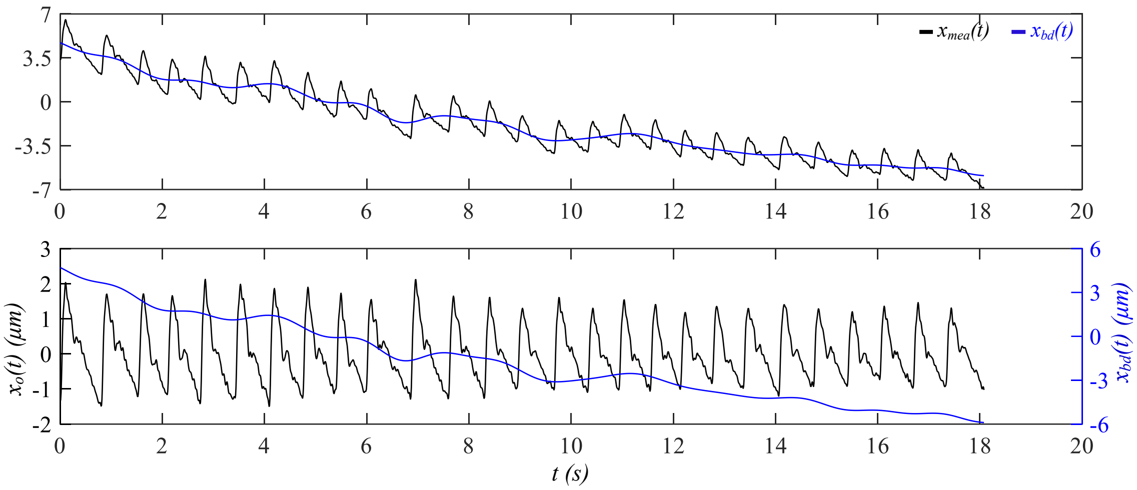

Figure S7. 5min post-exercise

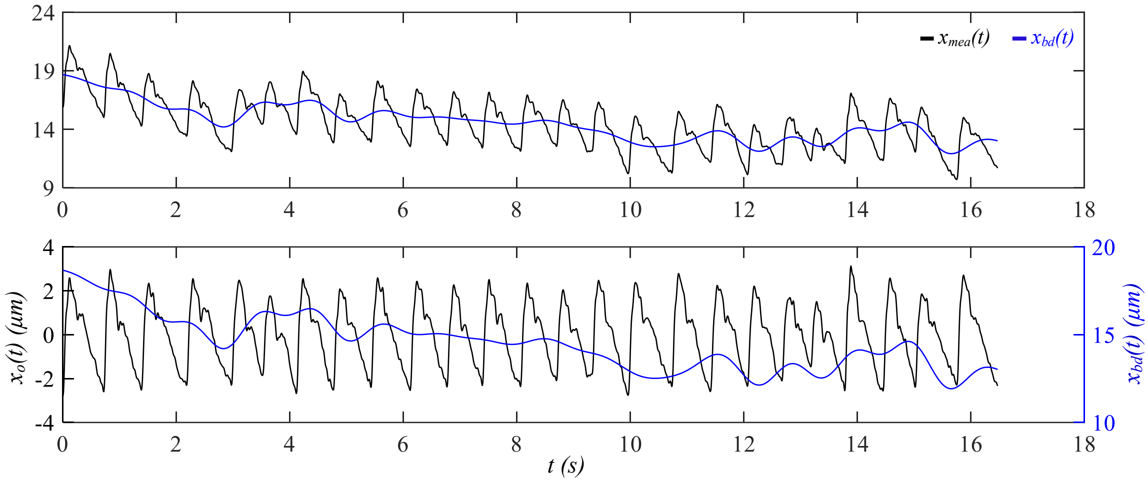

Figure S8. 10min post-exercise

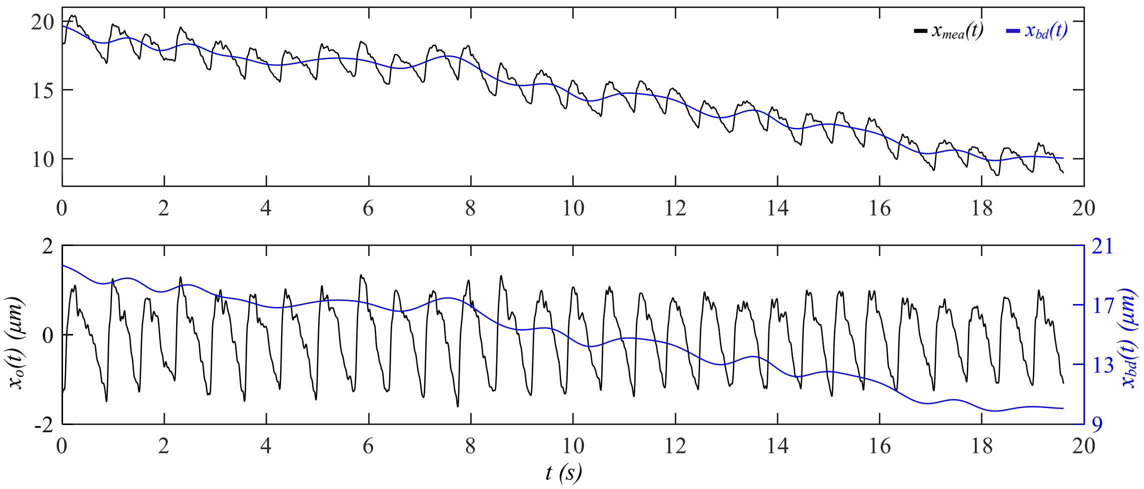

Figure S9. 20min post-exercise

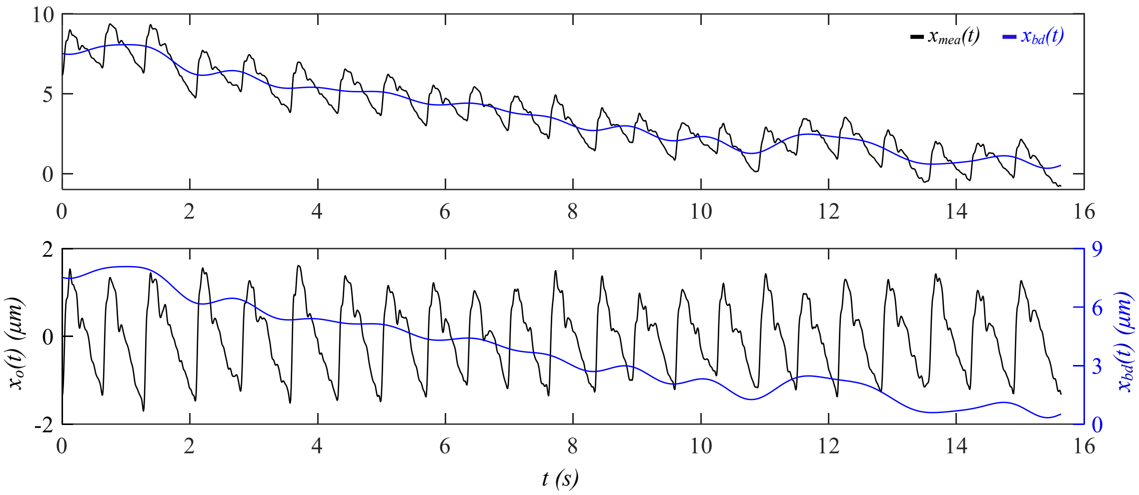

Figure S10. 30min post-exercise
